# Epidemiology and Diversity of Paratuberculosis in the Arabian Peninsula: A Systematic Review and Meta-Analysis with Implications for One Health

**DOI:** 10.3390/pathogens14090841

**Published:** 2025-08-23

**Authors:** Md Mazharul Islam, Ahmed K. Elfadl, Aisha Naeem, Randa Abdeen, Haya M. Al-Hajri, Md Abu Sayeed, Haileyesus Dejene, John I. Alawneh, Mohammad Mahmudul Hassan

**Affiliations:** 1 Department of Animal Resources, Ministry of Municipality, Doha, Qatar; ahmedpath@hotmail.com (A.K.E.); rahassan@mm.gov.qa (R.A.); hmai-hajri@mm.gov.qa (H.M.A.-H.); 2 Research and Graduate Studies, QU Health, Qatar University, Doha P.O. Box 2713, Qatar; a.naeem@qu.edu.qa; 3 National Centre for Epidemiology and Population Health (NCEPH), College of Health and Medicine, The Australian National University, Canberra, ACT 2601, Australia; abu.sayeed@anu.edu.au; 4 School of Veterinary Science, The University of Queensland, Gatton, QLD 4343, Australia; h.legese@uq.edu.au; 5 Plant Biosecurity and Product Integrity, Biosecurity Queensland, Department of Primary Industries, Brisbane, QLD 4000, Australia; john.alawneh@daf.qld.gov.au; 6 Faculty of Veterinary Medicine, Chattogram Veterinary and Animal Sciences University, Chattogram 4225, Bangladesh

**Keywords:** Johne’s disease, *Mycobacterium avium* subspecies *paratuberculosis*, prevalence, risk factors, genetic diversity, ruminants

## Abstract

Paratuberculosis is a chronic zoonotic bacterial infection, primarily affecting ruminants. This review examines the disease in the Arabian Peninsula, focusing on distribution, molecular diversity, prevalence, and associated risk factors. Following PRISMA guidelines, a systematic search was conducted in PubMed, Scopus, and Web of Science. After duplicate removal and eligibility screening, data extraction, analysis, and quality assessment were performed. Pathogen sequences were retrieved from NCBI GenBank for phylogenetic analysis. The review included a total of 31 published articles from 1997 to 2025, of which 26 were used in the meta-analysis. Most studies (n = 12) were published between 2011 and 2015, predominantly from Saudi Arabia (n = 22), with no reports from Qatar, Bahrain, or Yemen. The majority of the studies involved camels and sheep (n = 16 on each species), followed by cattle (n = 9), goats (n = 7), humans (n = 2), and buffalo (n = 1). Phylogenetic analysis delineates two major clades—Type S and Type C—suggesting greater genetic diversity in Type S. The estimated pooled seroprevalence and pathogen prevalence in livestock ruminants were 8.1% and 22.4%, respectively. Herd-level estimated pooled seroprevalence was 26.9%. Small ruminants (19.3%) were more sero-prevalent than large ruminants (7.4%), with goats (28.7%) significantly (*p* < 0.01) more affected than sheep (21.5%), camel (9.8%), and cattle (6.6%). Clinical signs in ruminants included chronic diarrhea, emaciation, anorexia, alopecia, wry neck, and dehydration. The reviewed study patterns and findings suggest high pathogen diversity and a significant risk of transboundary transmission at the human–animal interface in this region. A One Health surveillance approach is crucial, particularly on farms with diarrheic and emaciated animals. Establishing a national surveillance plan and phased (short-, intermediate-, and long-term) control programs is essential to mitigate economic losses, limit transmission, overcome the cultural barrier, and protect public health.

## 1. Introduction

Paratuberculosis, or Johne’s disease, is a chronic bacterial infection caused by *Mycobacterium avium* subspecies *paratuberculosis* (MAP). While it primarily affects ruminants such as cattle, sheep, and goats [1], it can also infect a wide range of species, including equids, camelids, carnivores, leporids, and even humans, raising significant zoonotic concerns. In ruminants, the disease follows a prolonged and progressive course, leading to severe weight loss (wasting), persistent diarrhea, and a marked decline in overall health and productivity [2,3]. Once clinical signs appear, bacterial shedding occurs primarily through feces, milk, and colostrum, with additional evidence suggesting possible shedding via semen and transplacental routes. The pathogen mainly transmits from one animal to another via the fecal-oral route through contaminated milk, feed, and water. However, transmission via aerosols (nasal route) and vertical (transplacental) transmission has also been documented [4,5,6]. The pathogen can survive for several months in the environment [7] and is resistant to conventional treatments. Free-living amoebae might act as reservoirs and vectors for the transmission of MAP [8]. Its insidious nature, long incubation period, and subclinical stages often delay diagnosis [2,3]. Severe emaciation, reduced milk production, and reproductive impairments contribute to its economic significance. Consequently, Johne’s disease is notifiable to the World Organization for Animal Health (WOAH) under the Terrestrial Animal Health Code [9,10].

In humans, MAP infection has been linked to irritable bowel syndrome and Crohn’s disease, with symptoms exacerbated in cases of co-infection [10,11]. First reported in Germany over a century ago, paratuberculosis is now globally distributed [10], including in the Arabian Peninsula [12], which comprises Bahrain, Kuwait, Oman, Qatar, Saudi Arabia (KSA), the United Arab Emirates (UAE), and Yemen, as well as parts of Iraq and Jordan. This region is widely recognized for its vast natural oil and gas reserves and holds strategic significance due to its highly diverse population, characterized by a large expatriate workforce, and its reliance on imported food supplies [13,14].

Traditional animal husbandry practices in the Arabian Peninsula, where multiple species of animals and birds are often kept together without proper biosecurity measures [15,16], may increase the risk of interspecies transmission of infectious diseases, including paratuberculosis. Therefore, preventive measures should be implemented to mitigate the transmission, reduce prevalence, and limit disease severity. While several studies have examined paratuberculosis in this region [12,17,18], no systematic review has comprehensively analyzed the disease. Thus, the present study aims to investigate its distribution, molecular characteristics, pooled prevalence, and associated risk factors in this region.

## 2. Materials and Methods

**Figure 1:**
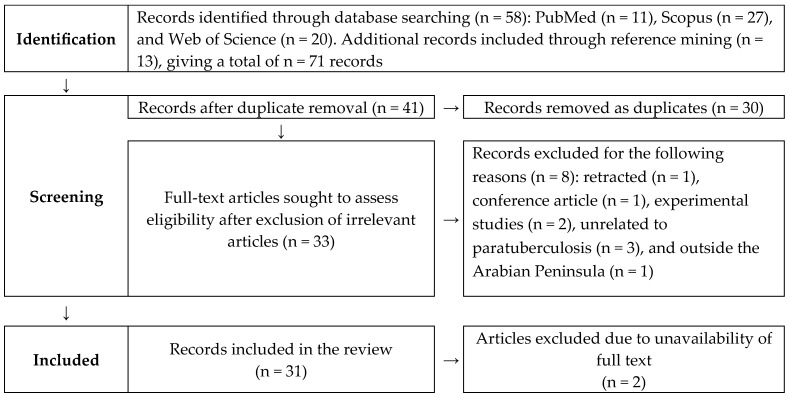
Systematic review PRISMA flow diagram describing the selection of published articles on paratuberculosis in the Arabian Peninsula in this study.

This systematic review was conducted in accordance with the PRISMA guidelines (Figure 1 and Supplementary Files S1–S3) [19,20,21]. The review protocol was registered in the Open Science Framework [22]. After the systematic search and removal of duplicates by one author, two authors independently conducted the eligibility screening, data extraction, quality assessment, after which their results were merged into a single document. One author then performed the data analysis. Any conflicts or uncertainties were discussed and resolved collectively.

### 2.1. Systematic Search

A literature search was conducted in PubMed, Scopus, and Web of Science on 10 January 2025, using the following keywords: (paratuberculosis OR “Johne’s disease” OR “*Mycobacterium avium* subspecies *paratuberculosis*” OR “*Mycobacterium paratuberculosis*”) AND (“Saudi Arabia” OR Qatar OR Kuwait OR Bahrain OR “United Arab Emirates” OR Yemen OR Oman OR Iraq OR Jordan). No restrictions were applied regarding the publication date or language.

The retrieved records were imported into EndNote 21 (Clarivate Analytics, Philadelphia, PA, USA) for duplicate removal. The remaining articles were screened by title and abstract using Rayyan (https://rayyan.qcri.org/), based on the following inclusion criteria: (1) studies reporting natural infections of paratuberculosis in any countries of the Arabian Peninsula; (2) any host species, including humans and animals; (3) study types including case reports, outbreak investigations, prevalence studies, or risk factor analyses. The exclusion criteria were the following: (1) conference proceedings and review articles; (2) studies unrelated to paratuberculosis; (3) articles published in languages other than English or Arabic; (4) studies from countries outside the Arabian Peninsula; and (5) retracted studies.

### 2.2. Data Extraction

Data extraction was conducted based on the availability of information in the included studies. The extracted variables included geographical data (sampling location and country), host-related data (species, gender, and age), laboratory findings (clinical feature, pathological findings, serological and pathogen detection results, and associated risk factors information) (Supplementary File S4).

### 2.3. Quality Appraisal

Each included study was assessed for the risk of bias in the following four key domains: selection bias, confounding bias, measurement bias, and data analysis bias. This assessment was performed using a 10-question appraisal tool [23], with responses classified as “yes”, “no”, “not applicable”, or “unclear”. A quality score (out of 100) was calculated as the percentage of “yes” answers out of the total “yes”, “no”, and “unclear” responses (excluding “not applicable”). Studies were then categorized as follows: high- (>70%), intermediate- (40–70%), and low-quality (<40%) articles [24].

### 2.4. Data Analysis

Descriptive statistics, including the number of articles, percentages, and 95% confidence intervals (CIs), were calculated using R software (RStudio, Version 2024.12.0+467). Meta-analysis and subgroup analysis were also conducted using the same software. For subgroup analysis, animals were categorized based on ruminant type (large ruminants: cattle and camels vs. small ruminants: sheep and goats), sex, and age. The pooled prevalence of paratuberculosis, along with the 95% confidence interval (CI) and *p*-value, was estimated using a random-effects model. Variations among the studies were assessed with chi-square (χ^2^) analysis and corresponding *p*-values, followed by the Inconsistency Index (*I*^2^) and Tau-squared (τ^2^) tests to quantify the degree of heterogeneity among studies and the variance among study effects, respectively. We assigned weights to each study based on the amount of information they provided. The results of the meta-analysis are presented using forest plots [25]. Funnel plots were also generated to evaluate potential publication bias by comparing the effect estimated against study size or precision. The geographical distribution of paratuberculosis in the Arabian Peninsula was mapped using RStudio.

### 2.5. Phylogenetic Analysis

The MAP IS900 gene was chosen for the phylogenetic analysis of the bacteria in this region based on the previous study [26]. After retrieving paratuberculosis gene sequences from NCBI GenBank, we performed the phylogenetic analysis using MEGA 11.0.13 (2022 release; Molecular Evolutionary Genetics Analysis software; Pennsylvania State University; available at https://www.megasoftware.net) [27], excluding sequences that lacked information about the host or sample collection time. The evolutionary history was inferred using the maximum likelihood method and the Tamura–Nei model [28].

## 3. Results

### 3.1. Research on Paratuberculosis in the Arabian Peninsula

**Figure 2:**
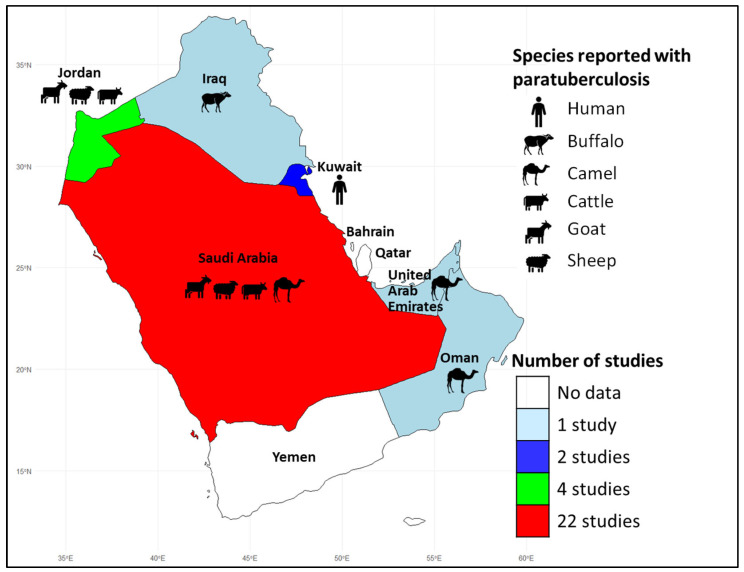
Map of the Arabian Peninsula showing the total number of studies included in this systematic review and animal species reported to have paratuberculosis.

The review included a total of 31 articles published between 1997 and 2025 (Table 1). Notably, the majority were published between 2011 and 2015 (n = 12, 38.71%, 95% CI: 22.42–57.71) and originated from Saudi Arabia (n = 22, 70.97%, 95% CI: 51.76–85.11) (Figure 2). No studies were found from Qatar, Bahrain, or Yemen. All articles were published in English, and no Arabic-language publications were identified. Most studies focused on camels and sheep, with 16 studies on each species (41.94%, 95% CI: 25.07–60.74).

Among the 31 articles, one was a case report, another was an editorial, and the remaining were cross-sectional studies. Of the cross-sectional studies, 15 (48.39%, 95% CI: 30.56–66.30) were rated as high quality, 11 (35.48%, 95% CI: 19.83–54.62) as intermediate quality, and 3 (9.68%, 95% CI: 2.53–26.90) as low quality (Supplementary File S4). Editorial, case report, and low-quality studies were excluded from the meta-analyses. Additionally, approximately 60% of the studies included in the meta-analysis fell outside of the funnel plots (Supplementary File S5).

### 3.2. Distribution and Evolutionary Diversity

Out of the eight countries in the Arabian Peninsula, five have reported the presence of the disease (Figure 2). In these countries, MAP has been identified in various livestock species, including buffalo, cattle, camels, goats, and sheep. Among ruminants, the disease was first documented in sheep in the Eastern Province of Saudi Arabia in 2000 [30]. In humans, however, the disease was reported only in Kuwait in 2019 [51].

**Figure 3:**
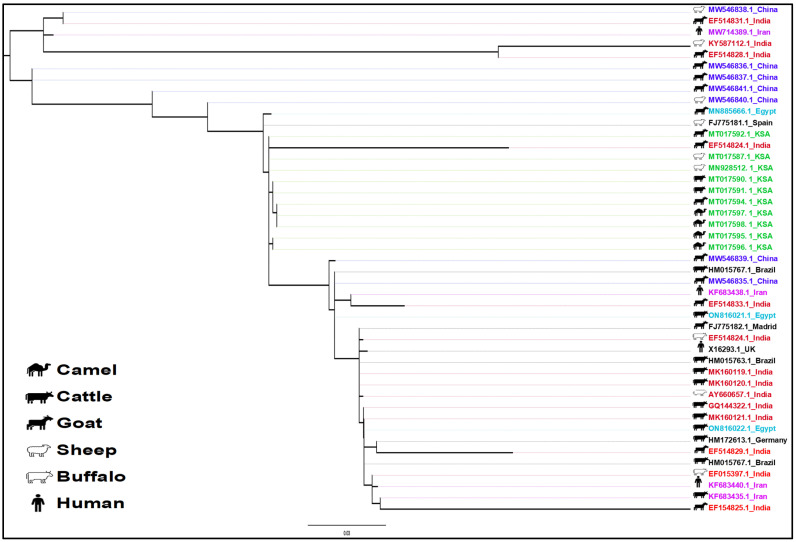
Phylogenetic tree showing the molecular relationship of the paratuberculosis sequences isolated from various animals in the Arabian Peninsula.

The phylogenetic analysis of the MAP sequences reveals a complex evolutionary history, host adaptation, and distribution pattern. The phylogenetic tree (Figure 3) delineates two major clades, corresponding to the Type S and Type C lineages of MAP. These clades suggest an ancient divergence, likely preceding modern animal husbandry practices. The higher diversity observed within the Type S clade suggests it may represent an older lineage, while Type C has likely emerged as a more recent, host-adapted variant. This clade is larger, with shorter branch lengths and higher genetic homogeneity among cattle- and camel-associated strains. In contrast, the Type S clade exhibits longer branches and fewer isolates, suggesting greater genetic diversity among sheep-associated strains. The isolates from Saudi Arabia are exclusively found in the Type C clade and are closely related to isolates from India, Egypt, and China. The sheep-associated strains are also closely related to isolates found in Spain and Brazil.

### 3.3. Prevalence

**Figure 4:**
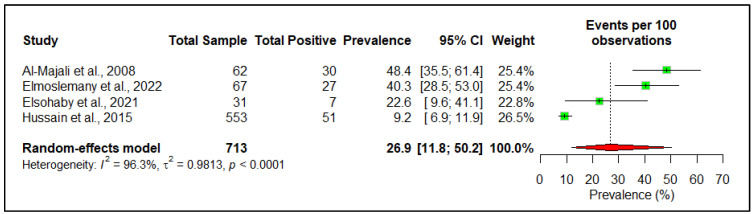
Livestock herd-level estimated pooled seroprevalence of paratuberculosis in the Arabian Peninsula [26,32,48,53].

**Figure 5:**
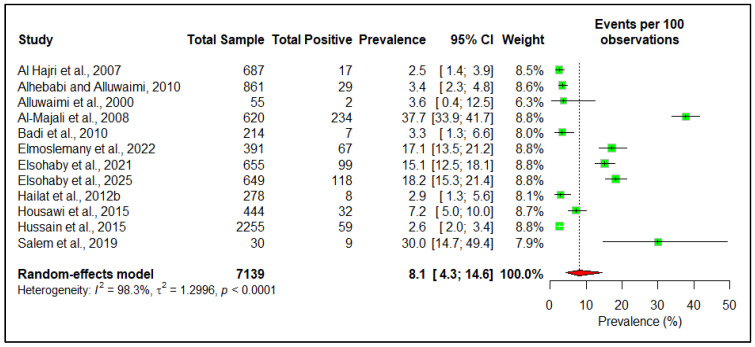
Individual animal-level estimated pooled seroprevalence of paratuberculosis in the Arabian Peninsula [17,26,30,31,32,33,35,44,47,48,53,54].

**Figure 6:**
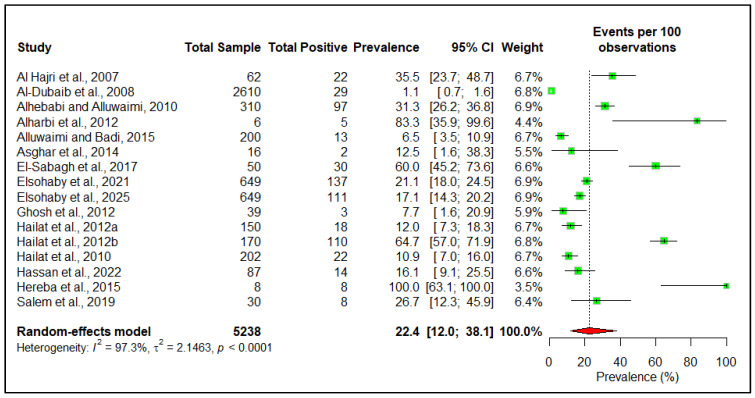
Individual animal-level estimated pooled pathogen prevalence of paratuberculosis in the Arabian Peninsula [12,17,26,33,36,37,38,39,41,43,44,45,46,50,54,55].

At the herd level, the estimated pooled seroprevalence is 26.9% (95% CI: 11.8–50.2) (Figure 4). However, at the individual animal level, the estimated pooled seroprevalence of the disease among livestock in the Arabian Peninsula is 8.1% (95% CI: 4.3–14.6) (Figure 5). The pooled pathogen prevalence among livestock is 22.4% (95% CI: 12.0–38.1) (Figure 6). Additionally, we analyzed the data based on different risk factors, including the country of origin, ruminant type, species, age, and sex, for both seroprevalence (Table 2) and pathogen prevalence (Table 3) (Supplementary File S5). The subgroup meta-analysis revealed that the seroprevalence of paratuberculosis is higher in small ruminants (19.3%, 95% CI: 8.5–38.3) than in large ruminants (7.4%, 95% CI: 4.4–12.2). Among individual species, the highest seroprevalence was observed in goats (28.7%, 95% CI: 11.9–54.7), followed by sheep (21.5%, 95% CI: 17.6–26.0), camels (9.8%, 95% CI: 6.0–15.7), and cattle (6.6%, 95% CI: 2.4–16.7).

### 3.4. Clinical Features

This review identified several studies describing the clinical features of paratuberculosis; however, these studies did not provide a comprehensive account of the disease. In ruminants, the most commonly reported features include chronic diarrhea, emaciation, anorexia, alopecia, wry neck, and dehydration [26,30,38,41]. These symptoms were well-documented in dromedary camels. In addition, one study reported intermittent fever [42], while another documented chronic and intermittent watery diarrhea, reduced milk production, and intermandibular edema in camels [46]. Infected animals showed no response to antimicrobial therapy [17]. In other farm animals, persistent diarrhea and significant weight loss were documented [26].

### 3.5. Pathology

Necropsy can reveal the emaciation by gelatinous atrophy of subcutaneous and visceral fats. The intestine shows granulomas on the serosal surface and mild-to-severe congestion in the mucosa, along with a highly thickened and corrugated mucous membrane forming transverse ridges [12,18,26,36,38], mainly in the ileam, extending to the colon and rectum [40,45,46,49]. Whitish granulomas in the hepatic tissue [46] and splenomegaly can also be observed [38].

Infected animals showed a significant increase in WBC and neutrophil count, along with a decrease in RBC, hemoglobin, and hematocrit [38,42]. Elevated levels of AST, ALT, ALP, GGT, GLDH, creatinine, Mg, bilirubin, and BUN, as well as decreased levels of TP, Alb, and glucose, have been noted in the sera of infected animals [38,40,42]. Oxidative stress biomarkers, such as SOD, CAT, and RGSH, were reduced, whereas lipid peroxidation increased significantly. The acute-phase proteins and proinflammatory cytokines, including APP, Hp, SAA, Fb, IL-1α, IL-1β, IL-6, IL-10, TNF-α, and IFN-γ, were also elevated [42].

Microscopically, the intestinal villi can be atrophied, appearing short, blunt, and distorted, with hyperactive goblet cells in the villi and the crypts of lieberkuhn containing mucin droplets [40]. The major microscopic lesions include epithelioid cell infiltration in the intestinal epithelium and mesenteric lymph nodes [12,38,40,43]. Granulomatous lesions may be observed [43]. Lesion severity ranged from mild to severe [45].

In mild cases, the lamina propria and submucosa can be mildly and loosely infiltrated with mononuclear cells, consisting primarily of lymphocytes, with a few macrophages and plasma cells. Occasionally, epithelioid giant cells can be observed [45]. In severe cases, extensive numbers of inflammatory cells, mainly macrophages, epithelioid cells, lymphocytes, and plasma cells, are present throughout the intestinal layers. Multinucleated Langhans and foreign body giant cells are scattered throughout the affected tissues [36,45]. The most severely affected lymph nodes are characterized by granulomas formation with mineralization [18,36,45,49].

The liver shows the following two types of granuloma: lepromatous type, characterized by aggregates of epithelioid cells surrounded by a fibrous capsule, and tuberculoid type, characterized by giant cell formation [12]. Lepromatous granulomas can also be seen in the white pulp of the spleen [38]. Acid-fast staining revealed acid-fast rods, appearing as aggregates or dispersed within the cytoplasm of macrophages in the intestine and other visceral organs [38,45].

Immunohistochemistry also diagnosed paratuberculosis in the infected tissues. Several studies found that about 60% of the suspected animals tested positive using the IHC method in this region [36,45]. CD4+, CD8+, CD25+, WC1+, CD11c+, CD14, and CX3CR1 markers can be used in IHC to detect the pathogen. However, the expression levels of these markers can vary among different organs, such as the ileum and mesenteric lymph nodes, as well as old and young animals [52].

## 4. Discussion

This study provides an updated and comprehensive overview of paratuberculosis in the Arabian Peninsula. Despite its zoonotic potential, only two human studies have been conducted in this region, with most research focusing solely on domestic ruminants. No published data exist on wildlife species, such as gazelles, oryx, and addax, despite their presence in captivity in this region [56,57]. The complete absence of research in some of the countries suggests that the disease remains largely underreported. In addition, Saudi Arabia contributes the majority of available studies, primarily focusing on camels, revealing a regionally imbalanced research landscape that underscores the need for broader surveillance.

Although MAP has long been suspected of contributing to several human diseases—particularly Crohn’s disease—its role remains inconclusive [58]. In Iran, MAP was detected in 71.2% of patients with irritable bowel syndrome (IBS) and 43.2% of non-IBS individuals [59]. Similarly, multiple human cases of paratuberculosis have been reported in Egypt [60]. While Crohn’s disease has been documented in the Arabian Peninsula, no studies have established a definitive link between MAP infection and Crohn’s disease in humans. The scarcity of human-focused studies in this region, with only a single reported case, underscores the need for further research into potential zoonotic transmission and public health implications.

Paratuberculosis is globally distributed, affecting both domestic and wild ruminants, as well as other species, like horses, pigs, rabbits, and foxes [10]. It imposes a significant economic burden on small ruminant production and the dairy industry [9,61]. Historical records (pre-2005) archived on the WOAH website indicate the presence of MAP in the region [62]. Our findings confirm that the disease is widespread across the Arabian Peninsula and genetically linked to global MAP strains (Type S and Type C), likely facilitated by extensive international animal trade [63]. Most countries in the region import live animals, including sheep, goats, cattle, and camels, from MAP-endemic areas, such as Iran, Egypt, Sudan, and Somalia [64,65,66]. Moreover, during the camel racing and breeding seasons, animals are frequently transferred among the countries of the Gulf Cooperation Council (GCC), with minimal quarantine protocols, suggesting that livestock trade networks play a substantial role in regional disease transmission [15].

Seroprevalence data from our study show that herd-level MAP infection rates are higher than those in sheep flocks in South Africa (3%) but lower than those reported in Germany (65%) and Italy (73.7%) [9]. Goat seroprevalence was also higher than in Chile (0.82%) and USA (1.4%) and yet lower than in West Indies (83%) [9]. In cattle, prevalence in Egypt (16.1%) slightly exceeded our findings, while Iran reported only 8% PCR-positive camels [65,66]. These differences may stem from geographic, climatic, and management variations; from differing laboratory protocols; or from variations in study designs [67]. Further comparative studies are needed to determine the drivers behind such variability.

Contrary to prior studies that suggest MAP infection is more common in large ruminants [68], our findings indicate significantly higher seropositivity in goats and sheep. This may be reflected in pathogen strains (Type C vs. Type S), animal population density, and management practices. Type C infects a range of species, while Type S is more specific to small ruminants [69,70], and intra-species transmission is likely intensified by larger, denser flocks and limited biosecurity. In contrast, better management practices in cattle and camel farms may explain their lower seroprevalence.

During the incubation period, serological tests may yield false-negative results due to low antibody production, especially in the early stage of infection. Some animals may develop natural resistance, remain subclinical carrier, and intermittently shed MAP, leading to discrepancies between antigen and antibody test results. As the disease progresses and clinical symptoms appear, animals typically test positive for both antibodies and antigens. Because of these diagnostic challenges, relying on serology or antigen testing alone may be insufficient for a definitive diagnosis. Moreover, a positive antibody or antigen test result in a single animal often indicates that others in the herd are also infected, regardless of an animal’s age, sex, or clinical status. A study in Egypt supports this, finding no significant association between MAP prevalence and factors like age and grazing habit [48]. However, diarrhea remains a key clinical indicator of advanced disease stage, and MAP testing should be prioritized in cases of chronic, treatment-resistant diarrhea. Given the low specificity and sensitivity of individual diagnostic tests, combining at least two complementary methods (e.g., ELISA and PCR) is advised for surveillance and outbreak investigations.

Globally, prevention and control of paratuberculosis rely on the following three basic pillars: test-and-cull strategies, farm management improvements, and vaccination [71]. Australia is an exemplary model where integrated national control—combining regular testing, herd management, and long-term vaccination—has substantially reduced MAP prevalence [71,72]. However, applying these strategies in the Arabian Peninsula is particularly challenging due to deep-rooted socio-cultural practices and diverse national contexts. In the Gulf Cooperation Council (GCC) countries, livestock are often raised in *Izbahs*, where multiple animal species and age groups housed together with minimal biosecurity measures. Emotional attachment to animals, especially high-value racing camels and breeding stocks, often prevents surveillance, investigation, and culling. *Majlis* gathering within farm compounds, shared accommodations between workers and animals, and raw milk consumption further compound transmission risks [15]. In contrast, countries like Jordan and Oman may be more amenable to structured interventions, while war-torn nations such as Iraq and Yemen pose logistical and political barriers to disease control. In all cases, traditional lifestyles and limited awareness complicate the application of conventional test-and-cull or depopulation strategies. Given these diverse socio-cultural, economic, and political realities across the Arabian Peninsula, a one-size-fits-all control approach is unlikely to succeed. Therefore, a phased strategy—tailored to national capacity and cultural feasibility—is essential. Short-, intermediate-, and long-term interventions can offer a flexible roadmap for regionally appropriate MAP control under a One Health framework.

Short-term (~2 year) control should focus on low-barrier, culturally appropriate interventions within the One Health framework [73,74]. Educational campaigns targeting farm owners, workers, and veterinarians should highlight MAP’s economic and public health impacts. Community-supported awareness programs should promote basic hygiene, such as separating young stock from adults and reducing fecal contamination. Confidential screening (e.g., ELISA and PCR) can identify high-shedding animals for isolation rather than culling. Public health messaging should discourage raw-milk consumption. Human health surveillance, particularly among individuals with close animal contact and IBS-like symptoms, should be integrated. Pilot vaccination programs in government farms or among cooperative private owners could provide valuable proof-of-concept data.

Intermediate-term (2–5 years) strategies should focus on building core infrastructure and initiating sustainable practices. These include piloting herd health programs in farms willing to adopt moderate biosecurity measures. National diagnostic guidelines standardizing ELISA and PCR should be implemented through regional veterinary laboratories. Animal identification and record-keeping systems will enable traceability and targeted interventions. Establishing regional reference laboratories with MAP testing capacity is essential for sustained surveillance and outbreak response. Incentive-based programs may increase farmer engagement and support for improvements in housing, feeding, and hygiene. One Health research should begin to explore MAP exposure in high-risk human populations. For herds where culling is not applicable, context-appropriate alternatives, such as long-term segregation of positive animals, should be promoted.

Long-term control will require coordinated, region-specific strategies that integrate veterinary, human health, and environmental sectors. Government-led incentives can encourage voluntary disease reporting, testing, and improved biosecurity. Guidelines should offer alternatives to culling of MAP-positive animals. In high-prevalence commercial herds, sanitary emptying and disinfection may be feasible. Strengthening veterinary services and infrastructure, along with comprehensive herd health and MAP surveillance programs, can serve as a regional model. Meanwhile, international support and humanitarian coordination are essential for any feasible public or animal health intervention in Iraq and Yemen.

However, this study has several limitations, including overrepresentation of data from Saudi Arabia and potential underreporting from countries, such as Bahrain, Qatar, Kuwait, and Yemen. Additionally, most studies relied on cross-sectional designs, limiting longitudinal interpretation and the assessment of risk-factor dynamics.

## 5. Conclusions

The current study provides an update on the distribution and prevalence, as well as associated risk factors of paratuberculosis in the Arabian Peninsula. Small ruminants showed a higher prevalence of the disease than large ruminants. Although the disease is well-known for its economic significance and zoonotic potential, it remains largely neglected in this region. To effectively address the disease, a One Health surveillance approach should be prioritized, particularly on farms with diarrheic and emaciated animals. Establishing a national One Health surveillance plan, developing reliable screening protocols, and implementing short-, intermediate-, and long-term control programs are essential for controlling the disease in this region.

## Figures and Tables

**Table 1:** Summary characteristics of the reviewed articles on paratuberculosis in the Arabian Peninsula.

Characteristics	Number of Articles (%, 95% CI)	References
Publication year		
1996–2000	2, 6.45 (1.12–22.84)	[29,30]
2006–2010	8, 25.54 (12.54–44.93)	[12,31,32,33,34,35,36,37]
2011–2015	12, 38.71 (22.42–57.71)	[38,39,40,41,42,43,44,45,46,47,48,49]
2016–2020	4, 12.90 (4.21–30.76)	[17,50,51,52]
2021–2025	5, 16.12 (6.09–34.47)	[18,26,53,54,55]
Animal species		
Buffalo	1, 3.23 (0.16 – 18.51)	[55]
Camel	16, 41.94 (25.07 – 60.74)	[17,18,26,33,34,38,40,42,43,46,47,48,50,52,53,54]
Cattle	9, 29.03 (14.89 – 48.24)	[26,31,35,37,39,44,45,51,54]
Goat	7, 22.58 (10.28–41.54)	[12,26,32,41,44,49,54]
Sheep	16, 41.94 (25.07 – 60.74)	[17,18,26,33,34,38,40,42,43,46,47,48,50,52,53,54]
Human	2, 6.45 (1.12–22.84)	[29,51]
Study detail		
Clinical history	20, 64.51 (45.38 – 70.17)	[12,17,18,26,29,30,31,34,37,38,40,41,42,43,46,47,48,50,51,53]
Pathological study	14, 48.28 (29.89–67.10)	[12,18,26,33,34,36,38,40,42,43,45,46,49,52]
Serology	15, 48.39 (30.56 – 66.60)	[17,26,30,31,32,33,34,35,39,45,47,48,51,53,54]
Pathogen detection	20, 64.51 (45.38 – 70.17)	[12,17,18,26,29,30,31,33,36,37,38,39,41,43,44,45,46,50,51,55]
Study type		
Cross-sectional	29, 93.35 (77.15–98.87)	[12,17,26,29,30,32,33,34,35,36,37,38,39,40,41,42,43,44,45,46,47,48,49,50,51,52,53,54,55]
Case report	1, 3.45 (0.1–19.63)	[18]
Editorial	1, 3.45 (0.1–19.63)	[31]

**Table 2:** Risk factors associated with animal-level seroprevalence of paratuberculosis in the Arabian Peninsula: meta-analysis of antibody-based studies.

Sl No.	Risk Factor	Variable	Number of Studies	Estimated Pooled Prevalence	95% Confidence Interval	Heterogeneity *I*^2^ (%)	*p*-Value
1	Country	Saudi Arabia	9	8.3	4.4–15.1	98	0.9
		Other countries *	3	7.4	1.0–37.5		
2	Type	Large ruminant	4	7.4	4.4–12.2	97	0.05
		Small ruminant	10	19.3	8.5–38.3		
3	Species	Camel	7	9.8	6.0–15.7	97	<0.01
		Cattle	5	6.6	2.4–16.7		
		Goat	3	28.7	11.9–54.7		
		Sheep	4	21.5	17.6–26.0		
4	Sex	Female	4	7.4	2.0–24.1	89	0.90
		Male	2	8.2	3.1–17.9		
5	Age	Adult	5	7.0	3.0–15.6	91	0.32
		Female	4	3.3	0.9–11.1		

* Jordan, Oman, and United Arab Emirates.

**Table 3:** Risk factors associated with animal-level pathogen prevalence of paratuberculosis in the Arabian Peninsula: meta-analysis of pathogen detection studies.

Sl No.	Risk Factor	Variable	Number of Studies	Estimated Pooled Prevalence	95% Confidence Interval	Heterogeneity *I*^2^ (%)	*p*-Value
1	Country	Saudi Arabia	12	23.1	10.2–44.3	97	0.68
		Jordan	3	24.0	5.2–64.3		
2	Ruminant type	Large ruminant	14	25.1	13.0–42.8	96	0.19
		Small ruminant	10	13.9	7.1–25.3		
3	Species	Camel	7	35.6	17.5–59.0	95	0.11
		Cattle	4	10.4	3.7–25.6		
		Goat	4	11.8	3.8–31.3		
		Sheep	5	11.2	3.1–33.2		
4	Sex	Female	4	32.4	16.2–54.3	71	0.72
		Male	2	48.3	2.9–96.7		
5	Age	Adult	6	23.5	6.8–56.6	97	0.33
		Young	6	45.2	17.4–57.1		

## Data Availability

All data were derived from publicly available sources and are included in the Supplementary Information.

## Supplementary Materials

The following supporting information can be downloaded at: https://www.mdpi.com/article/10.3390/pathogens14090841/s1, Supplementary File S1: PRISMA 2020 checklist; Supplementary File S2: PRISMA-S Checklist; Supplementary File S3: PRISMA 2020 for Abstracts Checklist; Supplementary File S4: Extracted data; Supplementary File S5: Forest and funnel plots.
