# Supplementary material for: Epidemiology and Diversity of Paratuberculosis in the Arabian Peninsula: A Systematic Review and Meta-Analysis with Implications for One Health
Source: Pathogens. 2025 Aug 23;14(9):841. doi: 10.3390/pathogens14090841 (PMC12472523; doi:10.3390/pathogens14090841)
Supplement: Supplementary file 1 [file pathogens-14-00841-s001.zip › Supplementary file S4_Extracted data.pdf]

Supplementary Table S1: Extracted data from the included articles

Sl No	Author Study type, location, time, and species	Clinical history/findings	Pathology	Serology or Antibody detection	Pathogen detection	Source, Quality, and other remarks
1	Ahmad and Mokaddas, 2019 [1]<br>Cross-sectional, Kuwait, and Humans	TB susptected human patients			Culture and PCR (pulmonary and extrapulmonary specimens)<br>Humans: 1033/1	Online<br>Quality: High (88%)
2	Al Hajri and Alluwaimi, 2007 [2]<br>Cross-sectional, Saudi Arabia (Central region and Eastern Province), Cattle	Apparently healthy cows		ELISA (serum)<br>Dairy cow: 687/17<br>1 Year: 173/1<br>2 Year: 158/3<br>4 Year: 155/7<br>6 Year: 201/6<br>Female: 687/17<br>Sample source: Farm: 687/17	PCR (Feces)<br>Dairy cow: 62/22<br>1 Year: 13/1<br>2 Year: 20/7<br>4 Year: 12/6<br>6 Year: 17/8<br>Female: 62/22<br>Sample source: Farm: 62/22	Online<br>Quality: High (100%)
3	Al Hajri and Alluwaimi, 2007 [3]<br>Cross-sectional, Saudi Arabia (Central region and Eastern Province), Cattle	Subclinically infected cows		ELISA (serum)<br>Dairy cow: 687/17<br>1 Year: 173/1<br>2 Year: 158/3<br>4 Year: 155/7<br>6 Year: 201/6<br>Female: 687/17<br>Sample source: Farm: 687/17	PCR (Feces)<br>Dairy cow: 62/22<br>1 Year: 13/1<br>2 Year: 20/7<br>4 Year: 12/6<br>6 Year: 17/8<br>Female: 62/22<br>Sample source: Farm: 62/22	Online. The editorial seems repetation of article no 2 [2]. Not used for meta-analysis and quality assesment.
4	Al-Dubaib and Mahmoud, 2008 [4]	Records of Qassim University indicated that each registered herd had at least on	Gross and histopathology<br>Gross, the affected animals exhibited enlarged mesenteric lymph nodes and a thickened, corrugated intestinal mucosa. Microscopically, the intestinal epithelium and mesenteric lymph nodes showed		ZN stain and Culture (Feces and tissue)<br>Herd: 90/18	Hand search<br>Quality: Intermediate (60%)

Sl No	Author Study type, location, time, and species	Clinical history/findings	Pathology	Serology or Antibody detection	Pathogen detection	Source, Quality, and other remarks
	Cross-sectional, Saudi Arabia (Qassim), Goats	convirmed case of Johne's disease.	infiltration by epithelioid cells. In the liver, two types of granulomas were observed: lepromatous granulomas, consisting of aggregates of epithelioid cells surrounded by a fibrous capsule, and tuberculoid granulomas, characterized by the presence of giant cells.		Goats: 2610/29<br>>3 Years: 2610/29<br>Breed Najdi: 2610/29<br>Sample source: Farm: 2610/29	
5	Al-Majali et al., 2008 [5]<br>Cross-sectional, Jordan (Tafelah and Ma'an)<br>May to August, 2007<br>Sheep and goats			ELISA (Serum)<br>Toal flock: 62/30<br>Sheep flock: 38/19<br>Goat flock: 24/11<br><br>Sheep: 320/71<br>Goat: 300/163<br>Sample source: Farm: 620/234		Online<br>Quality: High (70%)<br>Mixed farming and communal grazing are two major risk factors of MTB
6	Al-Rahman et al., 2020 [6]<br>Cross-sectional, Saudi Arabia (Eastern province); Camels		Immunohistochemistry:<br>Illeum:<br>In the ileum, CD3 expression was extensive in young normal camels, minor in old infected camels, and absent in young infected and old normal animals. CD4+ was strongly expressed in old normal camels but was undetectable in all other groups. CD8+ expression was observed only in old infected camels. CD25+ was widely expressed across all groups. WC1+ expression was high in young normal and old infected camels, moderate in old normal camels, and absent in young infected camels. CD11c+ was predominantly observed in infected camels, while CD14+ and CX3CR1 expression were specific to old infected camels. Madcam-1 expression was notable in all infected camels and present in old normal animals.<br>Mesenteric lymph node:			Rewrite the clinical features<br>Hand search<br>Quality: high (80%)<br>MTB prevalence was based on clinical examination, necropsy, ZN stain, and ELISA. Therefore, this result can not be segregated as antibody, antigen, or nucleotide detection. So, the data were not used in meta-analysis

Sl No	Author Study type, location, time, and species	Clinical history/findings	Pathology	Serology or Antibody detection	Pathogen detection	Source, Quality, and other remarks
			CD3+ cells were observed only in young normal and old infected camels. CD4+ was not detected in any group. CD8+, CD25+, WC1+, and CD11c+ were extensively expressed in all groups except young infected camels. CD14+ and CX3CR1 expression were restricted to young normal camels. Madcam-1 was broadly expressed in all groups except in the mesenteric lymph node of young infected camels.<br>Jejunum<br>In the jejunum, CD3+ expression was prominent in young normal and old infected camels, whereas CD4+ and CD8+ were not detected in any group. CD25+ was widely expressed in all groups. WC1+ (γδ cells) were abundant in all groups, primarily around submucosal blood vessels. CD11c+ was highly expressed in all groups except young normal camels. CD14+ was detected only in infected camels, with highest expression in young infected animals and moderate to low expression in other groups. CX3CR1 was mainly observed around the epithelial tissues of villi. Madcam-1 expression was high in all groups except old normal camels.<br>Sample source: Slaughterhouse			
7	Al-Shamali et al., 1997 [7]<br>Cross-sectional, Kuwait, Humans	Human patients of Chron's disease, ulcerative colitis, and IBS			PCR<br>Humans: 37/0	Hand search<br>Quality: high (83%)
8	Alhebabe and Alluwaimi, 2010 [8]<br>Cross-sectional, Saudi Arabia, Camel		Necropsy<br>Gross examination revealed that some camels had emaciated ileum and mesenteric lymph nodes, while others showed no pathological signs characteristic of Johne's disease.	Sample: Serum<br>Camels: 861/29<br>1-4 years: 386/14<br>5-9 years: 268/5	Culture and PCR (Feces)<br>Fecal culture: 310/0<br>PCR:<br>Camels: 310/97	Hand search<br>Quality assesment: High (83%)<br><br>Ziehl–Neelsen–stained smears from

Sl No	Author Study type, location, time, and species	Clinical history/findings	Pathology	Serology or Antibody detection	Pathogen detection	Source, Quality, and other remarks
				10-15 years: 207/10	1-4 years: 276/80<br>5-9 years: 23/12<br>10-15 years: 11/5	PCR-positive samples revealed moderate to densely concentrated numbers of M. paratuberculosis.Fecal culture from ELISA-PCR positive samples did not show any tangible growth of MAP colonies.
9	Alharbi et al., 2012 [9]<br>Saudi Arabia (Qassim)<br>Camels	Clinical features observed in the camels included chronic watery diarrhea, alopecia, emaciation, wry neck, and dehydration	Clinical pathology: Low hematocrit (HCT), along with elevated levels of aspartate aminotransferase (AST) and creatinine.<br>Gross pathology: Grossly, the intestines showed granulomas at the serosal surface, along with a highly thickened and corrugated mucous membrane. The livers revealed small whitish granulomas and diffuse greyish-brown discoloration. Splenomegaly was observed without visible granulomatous lesions in the spleen.<br>Microscopic pathology: Histopathological examination revealed extensive infiltration of epithelioid cells (macrophages) in the mucosa and submucosa, with acid-fast bacilli present. Lepromatous granulomas were identified in the liver and the white pulp of the spleen. The mesenteric lymph nodes were enlarged and contained greyish granulomas, and similar granulomatous lesions were observed in the hepatic lymph nodes. Lepromatous granulomas in the lymph nodes also showed acid-fast bacilli upon tissue sectioning and scraping. Acid-fast bacilli were detected within macrophages across all examined organs.		PCR (tissue)<br>Camels (3-5 years): 6/5<br>Hospital: 6/5	Online<br>Quality: High (100%)

Sl No	Author Study type, location, time, and species	Clinical history/findings	Pathology	Serology or Antibody detection	Pathogen detection	Source, Quality, and other remarks
10	Alluwaimi and Badi, 2015 [10] Cross-sectional, Saudi Arabia (Qassim), Cattle			ELISA<br>Dairy cows (Feces): 200/13	PCR and Microarray<br>Dairy cows (Feces): 200/13	Hand search<br>Low (33%)
11	Alluwaimi et al., 2000 [11] Cross-sectional, Saudi Arabia (Eastern province) Sheep	Clinical history: The majority of the sheeps were showing diarrhea and emaciation<br>Diarrhea and emaciation: 55/29<br>Diarrhea: 55/9<br>Emaciation: 55/5<br>Apparently healthY: 55/12		Gel immunodiffusion test in serum: 55/2	Zeihl-Neelsen stain on fecal smears: 55/21	Hand search<br>Quality: High (83%)
12	Alluwaimi, 2008 [12] Cross-sectional: Saudi Arabia (Dammam) Camel	Clinical feature: Only a single emaciated camel showed signs of being ELISA positive.	Post-mortem examination of the ileum and mesenteric lymph nodes from emaciated camels revealed no significant histological changes.	ELISA (Serum)<br>Meat camel: 95/8<br>2-3 years: 3/0<br>5-6 years: 21/0<br>7-9 Years: 24/2<br>10-15 Years: 47/6<br>Sample source: Slaughterhouse: 98/8		Online<br>Quality: Low (30%)
13	Almujalli and Ghamdi, 2012 [13] , Cross-sectional, Saudi Arabia (Eastern province), Camel	The animals had the clinical signs of watery diarrhea, weakness, emaciation, fluctuating	Camels 2-4 years): 12/12<br>Clinical pathology: Significant increase of granulocytes, leukocytes, and decrease of hemoglobin, RBCs, and Hct. Creatinine, Mg, AST, ALT, and BUN were increased, where TP and Alb were decreased.<br>Gross pathology:			Hand search<br>Quality: Low (0%)

Sl No	Author Study type, location, time, and species	Clinical history/findings	Pathology	Serology or Antibody detection	Pathogen detection	Source, Quality, and other remarks
		temperature, and dehydration.	Lesions were predominantly observed in the ileum and colon, characterized by thickening and corrugation of the intestinal wall, with the mucosa forming transverse folds. The ileocaecal and mesenteric lymph nodes were enlarged and edematous.<br><br>Microscopic pathology:<br>The intestinal villi appeared shortened, blunt, and distorted, with hyperactive goblet cells. The crypts of Lieberkühn contained abundant mucin droplets. The lamina propria was heavily infiltrated with mononuclear cells, primarily macrophages.Sample source: Hospital: 12/12			
14	Asghar et al., 2014 [14]<br>Cross-sectional: Saudi Arabia (Mekkah), November 2013 to February 2014; Sheep and goats	Clinical history: The animals exhibited signs of severe weakness and extreme thinness. Despite treatment with anthelmintics and antibiotics, the sick animals remained unresponsive, displaying intermittent diarrhea or soft, pasty stools, mild fever, lethargy, and depression.			Method: Zeihl-Neelsen stain and PCR on fecal sample<br>Sheep: 12/1<br>Goats: 4/1<br>Farm: 16/2	Hand search<br>Quality: Intermediate (44%)
15	Badi et al., 2010 [15]			Method: ELISA (serum)<br>Dairy cattle: 214/7		Hand search<br>Quality: Intermediate (60%)

Sl No	Author Study type, location, time, and species	Clinical history/findings	Pathology	Serology or Antibody detection	Pathogen detection	Source, Quality, and other remarks
	Cross-sectional, Saudi Arabia (Al-Ahsa), Cattle					Based on the objective of the review, the other methods were not considered in data extraction
16	El-Deeb et al., 2014 [16] Cross-sectional, Saudi Arabia (Eastern province), Camel	Clinical signs observed in the affected camels included persistent diarrhea, anorexia, and weight loss, along with weakness, emaciation, fluctuating body temperature, and dehydration	Clinical pathology:<br>In infected camels, there was a significant reduction in total erythrocyte count (TEC) and hemoglobin (Hb), accompanied by a marked increase in total leukocyte count (TLC), packed cell volume (PCV), and neutrophil percentage. Serum analysis revealed significant decreases in total proteins, albumin, and glucose compared to control animals, along with elevated levels of blood urea nitrogen (BUN) and bilirubin. Enzymatic activities of AST, ALT, ALP, GGT, and GLDH were significantly increased in infected camels.<br><br>Oxidative stress biomarkers:<br>Activities of superoxide dismutase (SOD) and catalase (CAT), as well as reduced glutathione (GSH) levels, were significantly decreased in infected camels, whereas lipid peroxidation was elevated, as indicated by higher malondialdehyde (MDA) concentrations.<br><br>Acute phase proteins and proinflammatory cytokines:<br>Serum levels of acute phase proteins, including haptoglobin (Hp), serum amyloid A (SAA), and fibrinogen (Fb), were significantly increased. Additionally, all measured pro-inflammatory cytokines—IL-1α, IL-1β, IL-6, IL-10, TNF-α, and IFN-γ—showed significant elevation in paratuberculosis-infected camels compared to controls.			Online<br>High (71%

Sl No	Author Study type, location, time, and species	Clinical history/findings	Pathology	Serology or Antibody detection	Pathogen detection	Source, Quality, and other remarks
17	El-Sabagh et al., 2017 [17]<br>Cross-sectional, Saudi Arabia (Al-Ahsa)<br>Camels	History of diarrhea			ZN stain: 3-5 months: 50/5<br>PCR: 3-5 Years: 50/30	Online<br>Quality: Intermediate (44%)
18	Elmoslemany et al., 2022 [18]<br>Cross-sectional, Saudi Arabia (Eastern province)<br>March 2014 to March 2015;<br>Camels			Method: ELISA<br>Serum<br>Camels: 391/67<br>Camel herds: 67/27.<br><br>Herd size, calving pen, and age of the camels are important risk factors. Higher herd, having no calving pen, and young camels were found to be more prevalent		Online<br>Quality: High (100%%)
19	Elsohaby et al., 2021 [19]<br>Cross-sectional, Saudi Arabia (Eastern province)<br>January – December 2019;<br>Camels, cattle, sheep, goats	The affected camels exhibited chronic diarrhea and significant weight loss	Necropsy showed thickened, corrugated ileum and enlarged, edematous mesenteric lymph nodes.	Method: ELISA<br>Camels (>2 Years): 240/22<br>Cattle (>2 Years): 66/13<br>Sheep (>2 Years): 220/43<br>Goats (>2 Years): 123/21<br>Herd: 31/7	Method: PCR<br>Camels (>2 Years): 240/24<br>Cattle (>2 Years): 66/20<br>Sheep (>2 Years): 220/59<br>Goats (>2 Years): 123/34<br>Herd: 31/23	Online<br>Quality: High (100%)

Sl No	Author Study type, location, time, and species	Clinical history/findings	Pathology	Serology or Antibody detection	Pathogen detection	Source, Quality, and other remarks
20	Elsohaby et al., 2025 [20] Cross-sectional, Saudi Arabia (Eastern province), January to December 2019.			ELISA (Serum)<br>Total: 649/118<br>Camels: 240/22<br>Sheep: 220/57<br>Goats: 123/25<br>Cattle: 66/14	PCR<br>Total: 649/16<br>Farm: 659/16<br>Camels: 240/26<br>Sheep: 220/62<br>Goats: 123/23<br>Cattle: 66/0	Fecal sample with ZN stain was also done, which was not included in the review and meta-analysis
21	Ghosh et al., 2012 [21] Cross-sectional, Saudi Arabia (Qassim & Al-Ahsa), Camels	Clinical feature: Chronic diarrhea and emaciation. Acid fast organism in feces	Histopathology: Granulomatous leions (mainly lymphocytic infiltration, macrophages, a few giant cells), covered with acid fast bacilli.		ELISA: 6/2 (Ag)<br>Culture: 39/3	Hand search
22	Hailat et al., 2012 [22] Cross-sectional, Jordan July 2007 to January 2008 Sheep, goat, and cattle				PCR (feces)<br>Slaughterhouse: 150/18<br>Sheep (Awassi): 50/9<br>Goat (Baladi): 50/5<br>Cattle (Friesian): 50/4	Online<br>Quality: Intermediate (44%)<br>Culture (feces)<br>Sheep (Awassi): 50/ 1<br>Goats (Baladi): 50/2<br>Cattle (Friesian): 50/2
23	Hailat et al, 2012 [23] Cross-sectional, Jordan, May to October 2007 Cattle	Clinical feature: poor to emaciation with no history of chronic diarrhea. About 8% of the suspected cattles were emaciated without any history of chronic diarrhea.	Gross pathology:<br>Examination of cattle intestinal segments, primarily the ileum, revealed mucosal thickening with corrugation in 7% of the animals. In three cases, prominent, white, enlarged, tube-like lymphatic vessels were observed in the intestine. Additionally, 5% of ileal and ileocecal mesenteric lymph nodes were enlarged to five- to sevenfold their normal size, accompanied by noticeable edema and congestion.<br><br>Histopathology:	ELISA (serum)<br>Cattle: 278/8	ZN feces: (278/8)<br>IHC: 170/110	Online<br>Quality: Intermediate (60%)

Sl No	Author Study type, location, time, and species	Clinical history/findings	Pathology	Serology or Antibody detection	Pathogen detection	Source, Quality, and other remarks
			Lesions varied from mild to severe (n = 263/62). In mild cases, the lamina propria and submucosa of the ileum showed loose infiltration of mononuclear cells, mainly lymphocytes, with occasional macrophages and plasma cells. Eosinophilic infiltrates and occasional degenerated or dead nematodes were observed. Epithelioid giant cells were rare. In severe cases, extensive infiltration of inflammatory cells—including macrophages, epithelioid cells, lymphocytes, and plasma cells—was observed throughout the intestinal layers. Multinucleated Langhans and foreign body giant cells were scattered throughout the mucosa and submucosa. Mild to severe granulomatous lymphangitis was prominent. Significant disruption of villous architecture, villous atrophy, and fusion were evident, while areas of necrosis were rare.<br><br>Special staining and immunohistochemistry:<br>Acid-fast bacilli staining revealed acid-fast rods, either aggregated or dispersed within the cytoplasm of macrophages. Immunohistochemistry confirmed infection in 170 of 110 samples (65%).			
24	Hailat et al., [24] Cross-sectional, Jordan, August to December 2001; Sheep (8-24month)	Apparently healthy sheep	Gross pathology:<br>The intestines and corresponding lymph nodes were examined grossly. Lesions ranged from mild changes to severe mucosal congestion and thickening of the ileal wall. In 13 animals, the ileal wall was markedly thickened with corrugation. In a few cases, the surrounding mesenteric lymph nodes were enlarged, edematous, and adhered to each other, forming cord-like structures due to tissue reactions.<br><br>Histopathology (n = 202/102):		ZN on histology sections: 202/22<br>ZN on direct smear: 202/ 53<br>Culture: 202/22<br>IHC: 134/124	Online<br>Intermediate (60%)

Sl No	Author Study type, location, time, and species	Clinical history/findings	Pathology	Serology or Antibody detection	Pathogen detection	Source, Quality, and other remarks
			Microscopic examination of H&E-stained ileal sections revealed variable mucosal thickening and congestion, primarily due to inflammatory cell infiltration. The mucosa and, less frequently, the submucosa were infiltrated by macrophages, lymphocytes, and plasma cells, with occasional epithelioid cells either scattered in the lamina propria and crypts or forming small nests. Multinucleated giant cells were rare. In some instances, cellular infiltrates were associated with caseous necrosis and/or mineralization. Peyer's patches frequently showed hyperplasia, with prominent "starry sky" tingible body macrophages. Granulomatous lymphangitis, with or without dilated lacteals, was commonly observed. Villi displayed changes including distortion, thickening due to inflammatory infiltrates, atrophy, and fusion.<br><br>Lymph node changes were characterized by lymphofollicular hyperplasia and mononuclear cell infiltration, predominantly epithelioid cells. Severely affected nodes exhibited granuloma formation with mineralization, while less affected nodes showed scattered epithelioid cells mixed with mononuclear cells and occasional neutrophils.<br><br>Immunohistochemistry:<br>Positive IHC reactions were indicated by brown staining around macrophage nuclei. In ileal samples, 134 of 124 sections were positive, while 123 of 72 mesenteric lymph node sections showed positive staining.*IHC of ileum (134/124).<br>*IHC of Mesentric LN (123/72).			

Sl No	Author Study type, location, time, and species	Clinical history/findings	Pathology	Serology or Antibody detection	Pathogen detection	Source, Quality, and other remarks
25	Hassan et al., 2022 [25]<br>Cross-sectional, Iraq (Masul, Ninevh governorate)<br>Buffalos				PCR and Culture<br>Buffalo fecal sample: 87/14<br>Herd: 12/4<br>Sample source: Farm	The first report of paratuberculosis in Iraq
26	Hereba et al., 2015 [26]<br>Cross-sectional, Saudi Arabia (Eastern Province)<br>Camels	The camels displayed chronic, intermittent watery diarrhea lasting 1–4 weeks, along with emaciation and reduced milk production; a few cases also showed intermandibular edema (bottle jaw).	Necropsy revealed emaciation and gelatinous atrophy of subcutaneous and visceral fat. Lesions were primarily observed in the ileum, extending to the colon and rectum, with marked thickening and corrugation of the small intestine and folding of the colonic mucosa. The ileocecal and mesenteric lymph nodes were congested and edematous, with some exhibiting granular changes. Whitish granulomas were also detected in the liver in three cases.		PCR<br>Camels: 8/8<br>Female: 5/5<br>Male: 3/5<br>4-6 Years: 8/8<br>Sample source: Hospital: 8/8	Online<br>Quality: High (100%)
27	Housawi et al., 2015 [27]<br>Cross-sectional, Saudi Arabia (Eastern province & Riyad)<br>January to March, 2010;<br>Camel			ELISA (serum)<br>Camels: 444/32<br>≤2 Years: 90/12<br>>2 Years: 254/20<br>Male: 98/5<br>Female: 346/27<br>Maghateer: 39/3<br>Sheal: 5/0<br>Soofer: 2/1<br>Hegen: 51/5<br>Magahem: 14/1<br>Riyadh: 101/10<br>Eastern region: 343/22		Online<br>Quality: High (100%)

Sl No	Author Study type, location, time, and species	Clinical history/findings	Pathology	Serology or Antibody detection	Pathogen detection	Source, Quality, and other remarks
28	Hussain et al., 2015 [28]<br>Cross-sectional, Oman (All nine governorates)<br>2009 to 2010;<br>Camels			ELISA (Serum)<br>Camels: 2255/59<br>Herd: 553/51		Online<br>Quality: High (100%)<br>Seroprevalence increse with age. Diarrhea in the herd is one of the factor to have higher seroprevalence of the disease.<br>Cattle are more seroprevalent than small ruminants
29	Radad and Khalil, 2011 [29]<br>Cross-sectional, Saudi Arabia (Jeddah)<br>Goats		Goats: 66/9<br>Grossly, goats with paratuberculosis exhibited significant emaciation. The intestinal mucosa, especially in the ileum, appeared thickened and corrugated, while the mesenteric lymph node was markedly enlarged. Upon cutting the mesenteric lymph nodes, multiple small granulomas with central caseation and slight calcification were observed.<br>Histologically, the duodenal and ileal villi appeared broader and blunter, infiltrated with lymphocytes. Additionally, the villi in these areas were heavily infiltrated with clusters of epithelioid cells. The mesenteric lymph nodes contained small to mid-sized granulomas primarily composed of epithelioid cells, featuring central necrosis and minimal calcification.			Online<br>Quality: Intermediate (60%)
30	Salem et al., 2019 [30]<br>Cross-sectional, Saudia Arabia<br>Camels<br>March 2014 to December 2015	Signs of chronic or intermittent diarrhea that are non-responsive to antibiotic treatment.		ELISA (Serum)<br>Camels: 30/9<br>ELISA<br>1-3 Years: 7/0<br>4-5 Years: 9/2<br>≥6Y: 14/7<br>Female: 18/7	Zeihl-Neelsen stain, PCR, Culture (Rectal/fecal samle)<br><br>Camel: 30/5 Zn	Hand search Quality: Intermediate (60%)

Sl No	Author Study type, location, time, and species	Clinical history/findings	Pathology	Serology or Antibody detection	Pathogen detection	Source, Quality, and other remarks
				Male: 12/2	1-3 Years: 7/0<br>4-5 Years: 9/0<br>≥6Y: 14/5<br>Female: 18/3<br>Male: 12/2<br><br>Camel: 30/8 PCR<br>1-3 Years: 7/0<br>4-5 Years: 9/0<br>≥6Y: 14/8<br>Female: 18/6<br>Male: 12/2<br><br>Camel: 30/5 Culture<br>1-3 Years: 7/0<br>4-5 Years: 9/0<br>≥6Y: 14/5<br>Female: 18/3<br>Male: 12/2	
31	Tigani et al., 2023 [31]<br>Case report, UAE (Abu Dhabi)<br>August 2017<br>Camel	Clinicl feature: Chronic watery diarrhea, dehydration, edematous swelling of ventral body sites, pica, and severe emaciation	Gross pathology:<br>The ileocecal region exhibited thickening and corrugation consistent with chronic proliferative enteritis. On sectioning, the ileal mucosa showed prominent folding and corrugation.<br><br>Microscopic pathology:<br>Histologically, the intestine displayed marked proliferative enteritis, characterized by stunted villi and lepromatous granulomas. These granulomas consisted of dense infiltrates of macrophages and epithelioid cells, along with lymphocytes in the mucosa and lamina propria, leading to significant		Zeihl-Neelsen stain and PCR (feces): Camels (female, 2 years): 1/1<br>Sample source: Hospital	Hand search Quality assessment was not considered and data were not included in the meta-analysis

Sl No	Author Study type, location, time, and species	Clinical history/findings	Pathology	Serology or Antibody detection	Pathogen detection	Source, Quality, and other remarks
			widening or thickening of the intestinal mucosa. Ziehl–Neelsen staining of serial intestinal sections revealed clusters of acid-fast bacilli.			

References

1. Ahmad, S.; Mokaddas, E. Diversity of nontuberculous mycobacteria in Kuwait: Rapid identification and differentiation of mycobacterium species by multiplex PCR, INNO-LiPA Mycobacteria v2 Assay and PCR Sequencing of rDNA. *Medical Principles and Practice* **2019**, *28*, 208-215, doi:10.1159/000498910.
2. Al Hajri, S.M.; Alluwaimi, A.M. The efficiency of ELISA and PCR in detecting subclinical paratuberculosis in the Saudi dairy herds. *Pakistan Journal of Biological Sciences* **2007**, *10*, 1906-1909, doi:10.3923/pjbs.2007.1906.1909.
3. Al Hajri, S.M.; Alluwaimi, A.M. ELISA and PCR for evaluation of subclinical paratuberculosis in the Saudi dairy herds. *Veterinary Microbiology* **2007**, *121*, 384-385, doi:10.1016/j.vetmic.2007.01.025.
4. Al-Dubaib, M.; Mahmoud, O. Paratuberculosis of goats at Qassim region of Central Saudi Arabia. *Bulgharian J. Vet. Med* **2008**, *11*.
5. Al-Majali, A.M.; Jawasreh, K.; Al Nsour, A. Epidemiological studies on foot and mouth disease and paratuberculosis in small ruminants in Tafelah and Ma' an, Jordan. *Small Ruminant Research* **2008**, *78*, 197-201, doi:10.1016/j.smallrumres.2008.05.012.
6. Al-Ramadan, S.; Al-Mohammed Salem, K.; Alshubaith, I.; Alluwaimi, A. CD markers of camel (Camelus dromedarius) intestine naturally infected with Mycobacterium avium subsp. paratuberculosis: Distinct expression of Madcam-1 and CX3CR1. *Turkish Journal of Veterinary and Animal Sciences* **2020**, *44*, 1010-1023, doi:10.3906/vet-2003-5.
7. Al-Shamali, M.; Khan, I.; Al-Nakib, B.; Al-Hassan, F.; Mustafa, A.S. A multiplex polymerase chain reaction assay for the detection of Mycobacterium paratuberculosis DNA in Crohn's disease tissue. *Scandinavian Journal of Gastroenterology* **1997**, *32*, 819-823, doi:doi:10.3109/00365529708996540.
8. Alhebabi, A.; Alluwaimi, A. Paratuberculosis in Camel (Camelus dromedarius): The Diagnostic Efficiency of ELISA and PCR. *The Open Veterinary Science Journal* **2010**, *4*, doi:10.2174/1874318801004010041.
9. Alharbi, K.B.; Al-Swailem, A.; Al-Dubaib, M.A.; Al-Yamani, E.; Al-Naeem, A.; Shehata, M.; Hashad, M.E.; Albusadah, K.A.; Mahmoud, O.M. Pathology and molecular diagnosis of paratuberculosis of camels. *Tropical Animal Health and Production* **2012**, *44*, 173-177, doi:10.1007/s11250-011-9905-2.
10. Alluwaimi, A.; Badi, F. Mycobacterium avium subspecies paratuberculosis infection in naturally infected cattle is associated with an upregulation of lipid metabolism gene expression. *International Journal on Advances in Life Sciences* **2015**, *8*, 377-397.
11. Alluwaimi, A.; Hatem, M.; Almousa, J. The efficacy of gel immunodiffusion and fecal smear tests for diagnosis of ovine paratuberculosis in sheep in Saudi Arabia. *Egyp J Immunol* **2000**, *7*, 29-32.
12. Alluwaimi, A.M. The efficiency of bovine elisa in detection of the mycobacterium avium subspecies paratuberculosis (MAP) infection in Camel (Camelus dromedaries) at different ages. *Journal of Camel Practice and Research* **2008**, *15*, 163-165.
13. Almujalli, A.M.; Ghamdi, G.M. Clinicopathological Findings of Partuberclosis in Camels Possible Steps for Control Strategy. *Research Journal of Biological Sciences* **2012**, *7*, 128-131, doi:10.3923/rjbsci.2012.128.131.
14. Asghar, A.; Abd El-Rahim, I.; Mohamed, A.; Ahmed, O. Clinical and molecular investigations of Johne's disease among small ruminants in Makkah, Saudi Arabia. *International Journal of Bioassays* **2014**, *3*, 3445-3451.
15. Badi, F.A.; Haroon, A.I.A.; Alluwaimi, A.M. The γδ cells as marker of non-seroconverted cattle naturally infected with Mycobacterium avium subspecies paratuberculosis. *Research in Veterinary Science* **2010**, *88*, 72-76, doi:doi:10.1016/j.rvsc.2009.06.004.

16. El-Deeb, W.M.; Fouda, T.A.; El-Bahr, S.M. Clinico-biochemical investigation of paratuberculosis of dromedary camels in Saudi Arabia: Proinflammatory cytokines, acute phase proteins and oxidative stress biomarkers. *Pakistan Veterinary Journal* **2014**, *34*, 484-488.
17. El-Sabagh, I.M.; Al-Ali, A.M.; Al-Naeem, A.A. Group A rotavirus and Mycobacterium avium subspecies paratuberculosis associated with diarrhea in dromedary camels in Eastern province, Saudi Arabia. *Thai Journal of Veterinary Medicine* **2017**, *47*, 383-388, doi:10.56808/2985-1130.2847.
18. Elmoslemany, A.; Alanazi, F.; Elsohaby, I.; Fayez, M.; Alnaeem, A. Associations between management factors and seroprevalence of Mycobacterium avium subsp. paratuberculosis in dromedary camels. *Comparative Immunology, Microbiology and Infectious Diseases* **2022**, *83*, doi:10.1016/j.cimid.2022.101780.
19. Elsohaby, I.; Fayez, M.; Alkafafy, M.; Refaat, M.; Al-Marri, T.; Alaql, F.A.; Al Amer, A.S.; Abdallah, A.; Elmoslemany, A. Serological and molecular characterization of mycobacterium avium subsp. Paratuberculosis (MAP) from sheep, goats, cattle and camels in the Eastern province, Saudi Arabia. *Animals* **2021**, *11*, 1-11, doi:10.3390/ani11020323.
20. Elsohaby, I.; Kostoulas, P.; Fayez, M.; Elmoslemany, A.; Alkafafy, M.E.; Bahhary, A.M.; Alzahrani, R.; Morsi, A.; Arango-Sabogal, J.C. Bayesian estimation of diagnostic accuracy of fecal smears, fecal PCR and serum ELISA for detecting Mycobacterium avium subsp. paratuberculosis infections in four domestic ruminant species in Saudi Arabia. *Vet Microbiol* **2025**, *301*, 110377, doi:10.1016/j.vetmic.2025.110377.
21. Ghosh, P.; Hsu, C.; Alyamani, E.J.; Shehata, M.M.; Al-Dubaib, M.A.; Al-Naeem, A.; Hashad, M.; Mahmoud, O.M.; Alharbi, K.B.J.; Al-Busadah, K.; et al. Genome-wide analysis of the emerging infection with mycobacterium avium subspecies paratuberculosis in the arabian camels (camelus dromedarius). *PLoS ONE* **2012**, *7*, doi:10.1371/journal.pone.0031947.
22. Hailat, N.; Fayyad, A.; Ababneh, M.; Hananeh, W.; Rezig, F.E.; Jaradat, S. PCR-restriction endonuclease analysis of Mycobacterium avium subsp. paratuberculosis isolates from goats, sheep, and cattle in Jordan. *Comparative Clinical Pathology* **2012**, *21*, 755-760, doi:10.1007/s00580-010-1170-7.
23. Hailat, N.; Hemida, H.; Hananeh, W.; Stabel, J.; Rezig, F.E.; Jaradat, S.; Al-Saleh, A.A. Investigation on the occurrence and pathology of paratuberculosis (Johne's disease) in apparently healthy cattle in Jordan. *Comparative Clinical Pathology* **2012**, *21*, 879-888, doi:10.1007/s00580-011-1192-9.
24. Hailat, N.Q.; Hananeh, W.; Metekia, A.S.; Stabel, J.R.; Al-Majali, A.; Lafi, S. Pathology of subclinical paratuberculosis (Johne's Disease) in Awassi sheep with reference to its occurrence in Jordan. *Veterinarni Medicina* **2010**, *55*, 590-602, doi:10.17221/2947-VETMED.
25. Hassan, A.A.; Rahawy, M.; Alkattan, L.M.; Khan, I.U.H.; Abdulmawjood, A.; Bülte, M. First report of paratuberculosis (Johne's disease) in livestock farms of river buffaloes (Bubalus Bubalis) in Nineveh, Iraq. *Veterinaria Italiana* **2022**, *58*, 231-236, doi:10.12834/VetIt.1866.9913.1.
26. Hereba, A.M.; Hamouda, M.A.; Al-Hizab, F.A. Pathological and molecular diagnosis of paratuberculosis among dromedary camels in Saudi Arabia. *Journal of Animal and Plant Sciences* **2015**, *25*, 997-1002.
27. Housawi, F.M.T.; Zaghawa, A.A.; Al-Naeem, A. Seroprevalence of paratuberculosis among camels in Al-Ahsa and Riyadh Regions, Kingdom of Saudi Arabia. *Pakistan Veterinary Journal* **2015**, *35*, 375-378.
28. Hussain, M.H.; Saqib, M.; Al-Maawali, M.G.; Al-Makhladi, S.; Al-Zadjali, M.S.; Al-Sidairi, T.; Asubaihi, S.; Al-Rawahi, A.; Mansoor, M.K. Seroprevalence of Mycobacterium avium subspecies paratuberculosis (MAP) and evaluation of risk factors in camels of the Sultanate of Oman. *Tropical Animal Health and Production* **2015**, *47*, 383-389, doi:10.1007/s11250-014-0734-y.

29. Radad, K.; Khalil, S. Coccidiosis, paratuberculosis and enterotoxaemia in saudi goats. *Brazilian Journal of Veterinary Pathology* **2011**, *4*, 219-224.
30. Salem, M.A.; El-Deeb, W.M.; Zaghawa, A.A.; Housawi, F.M.; Alluwaimi, A.M. Investigation of Mycobacterium paratuberculosis in Arabian dromedary camels (Camelus dromedarius). *Veterinary World* **2019**, *12*, 219-223, doi:10.14202/vetworld.2019.218-223.
31. Tigani, E.; Tigani-Asil, A.; El, G.; Abdelwahab, D.; Hadi, E.; Abdu, A.; Mohammed, A.; Terab, A.; Altaib, N.; Khalil, H.; et al. Pathological, microscopic, and molecular diagnosis of paratuberculosis/John's disease in naturally infected dromedary camel (Camelus dromedarius). *Veterinary World* **2023**, *16*, 1277–1283., doi:10.14202/vetworld.2023.1277-1283.
