# Supplementary material for: Epidemiology and Diversity of Paratuberculosis in the Arabian Peninsula: A Systematic Review and Meta-Analysis with Implications for One Health
Source: Pathogens. 2025 Aug 23;14(9):841. doi: 10.3390/pathogens14090841 (PMC12472523; doi:10.3390/pathogens14090841)
Supplement: Supplementary file 1 [file pathogens-14-00841-s001.zip › Supplementary file S5_Funnel and Forest plots.pdf]

Supplementary figures

1 Funnel plots

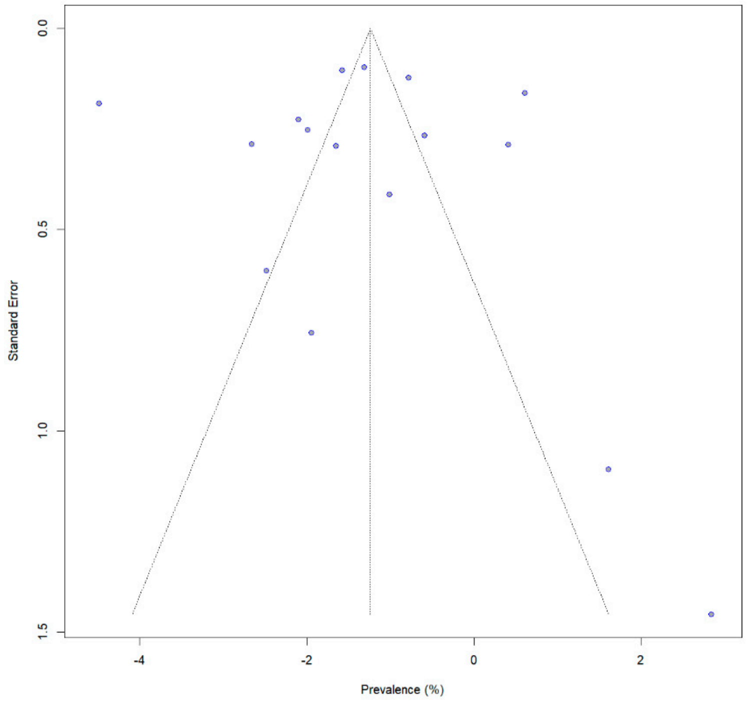

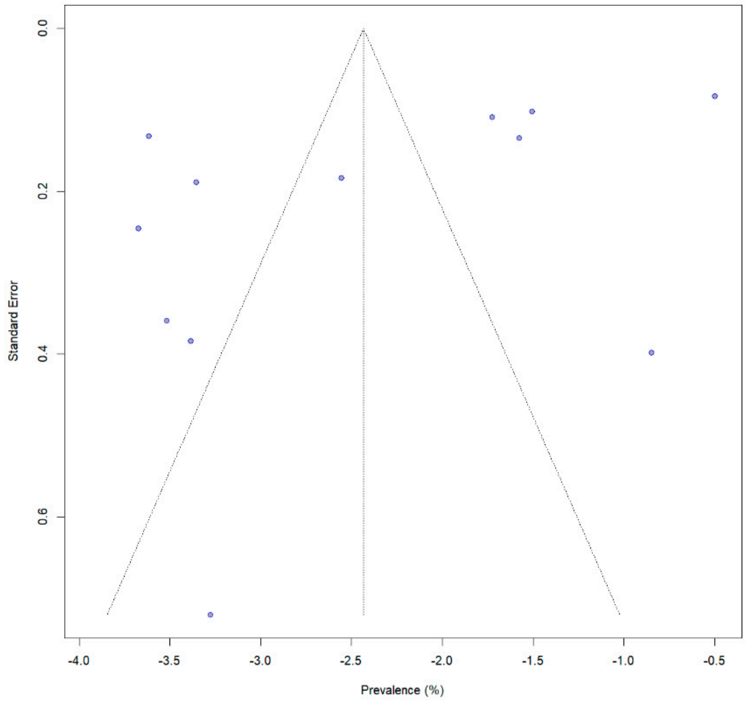

Supplementary Figure 1: Funnel plots showing seroprevalence (top) and pathogen prevalence (bottom) of paratuberculosis in livestock ruminants in the Arabian Peninsula.

2 Forest plots

2.1 Seroprevalence of paratuberculosis in ruminants

2.1.1 Herd level seroprevalence

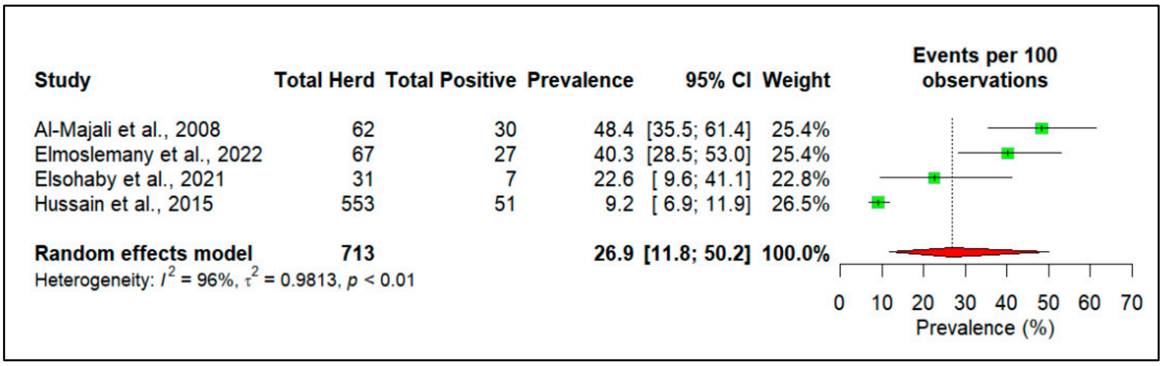

Supplementary Figure 2: Ruminant herd level seroprevalence of paratuberculosis in the Arabian Peninsula.

2.1.2 Individual level seroprevalence

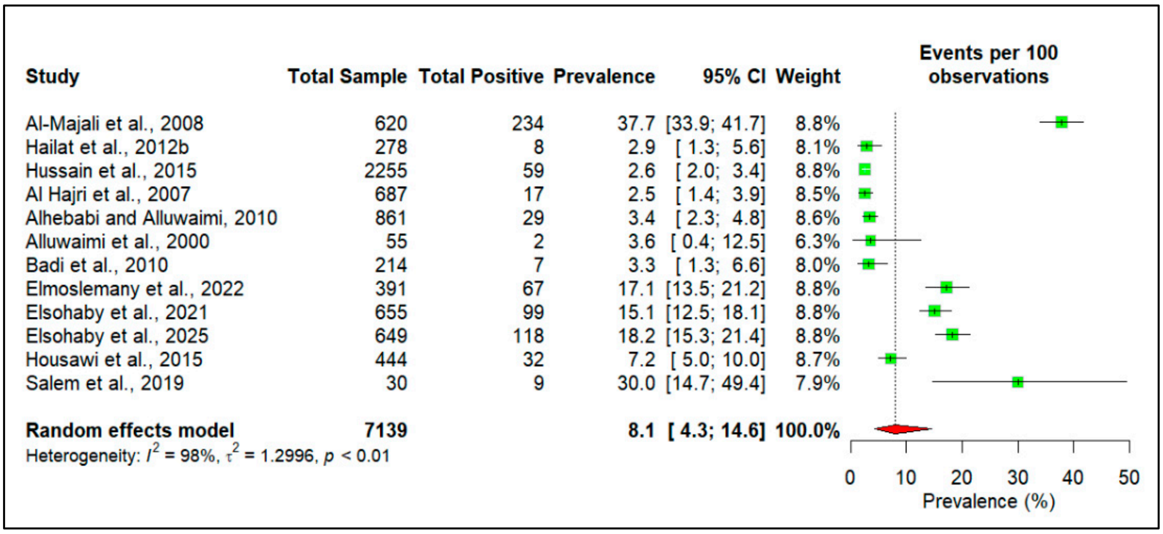

Supplementary Figure 3: Individual animal-level estimated pooled seroprevalence of paratuberculosis in the Arabian Peninsula.

2.1.3 Risk factors of seroprevalence

2.1.3.1 Country of origin

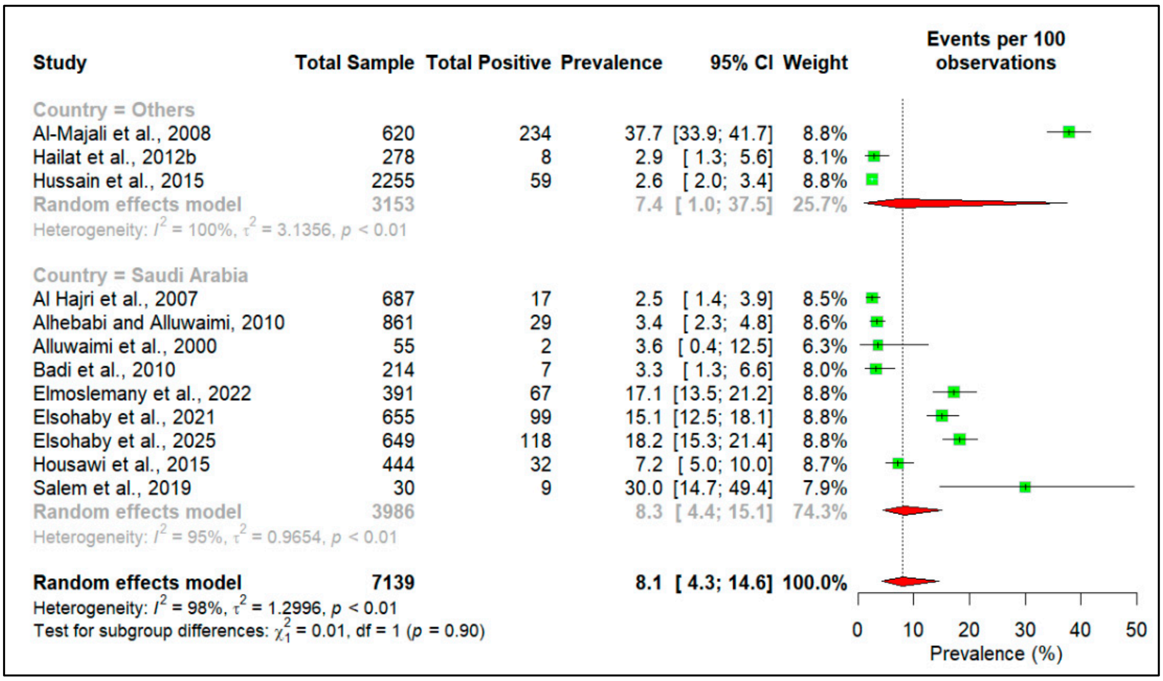

Supplementary Figure 4: Individual animal-level estimated pooled seroprevalence of paratuberculosis based on country of origin.

2.1.3.2 Species

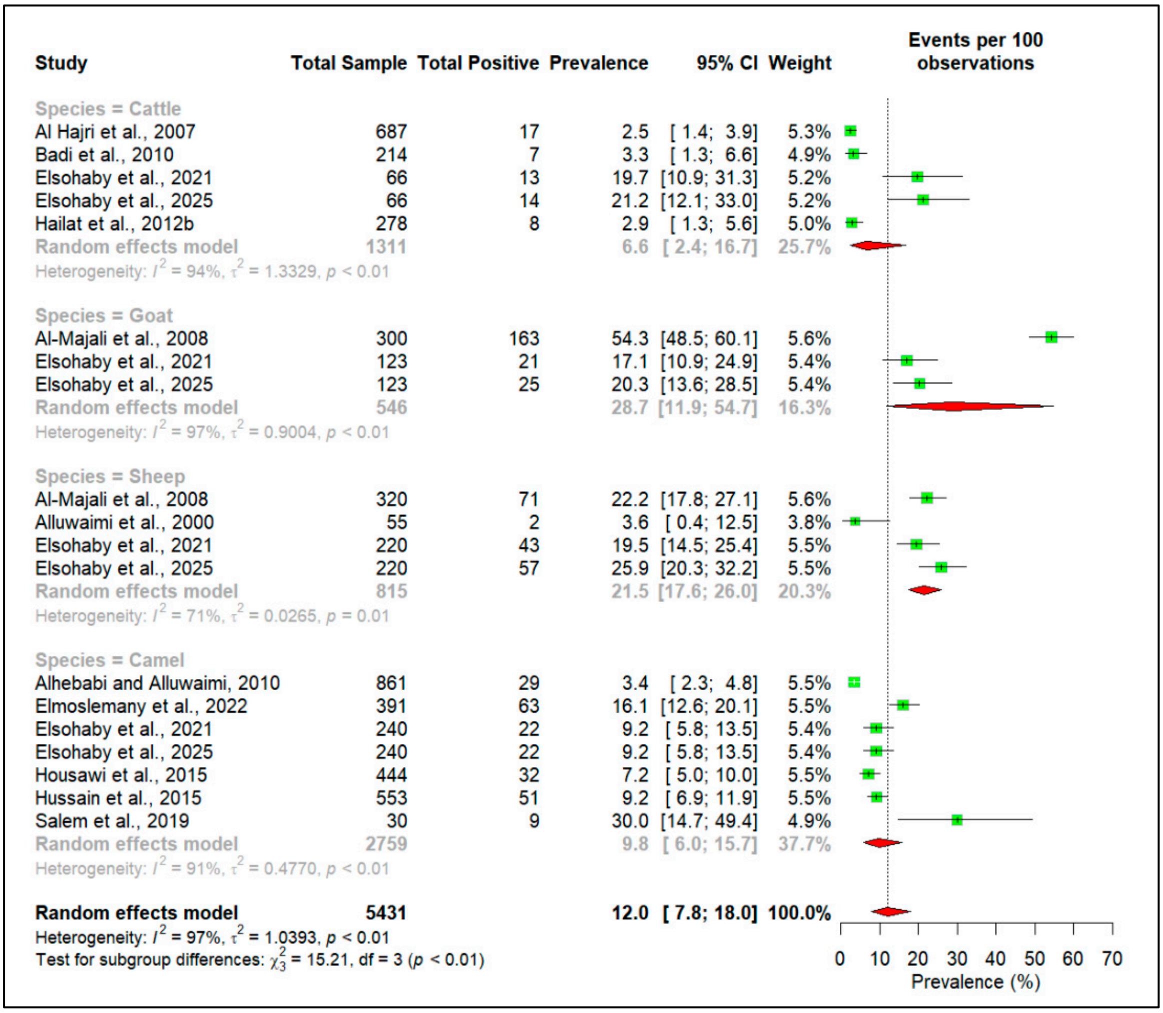

Supplementary Figure 5: Individual animal-level estimated pooled seroprevalence of paratuberculosis based on ruminant species.

2.1.3.3 Sex

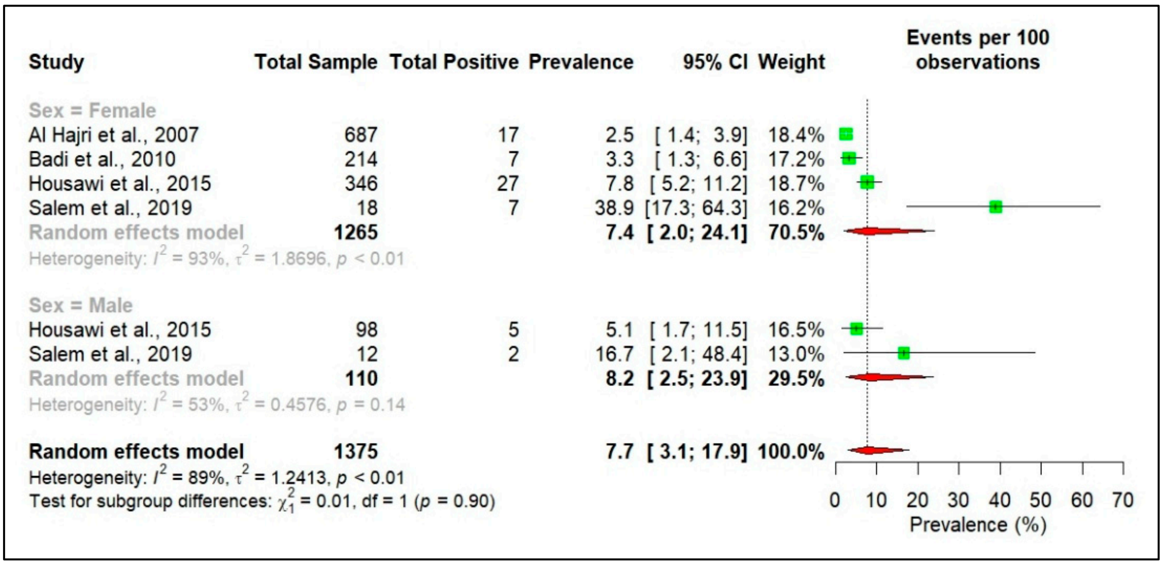

Supplementary Figure 6: Individual animal-level estimated pooled seroprevalence of paratuberculosis based on ruminant sex

2.1.3.4 Age

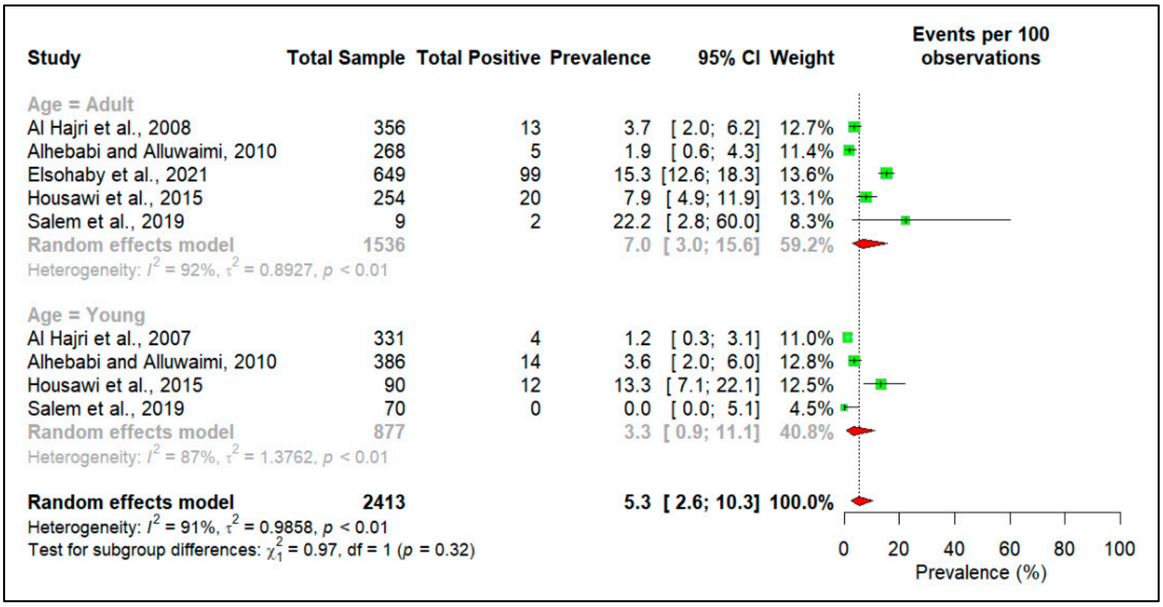

Supplementary Figure 7: Individual animal-level estimated pooled seroprevalence of paratuberculosis based on ruminant age.

2.2 Pathogen prevalence in ruminants

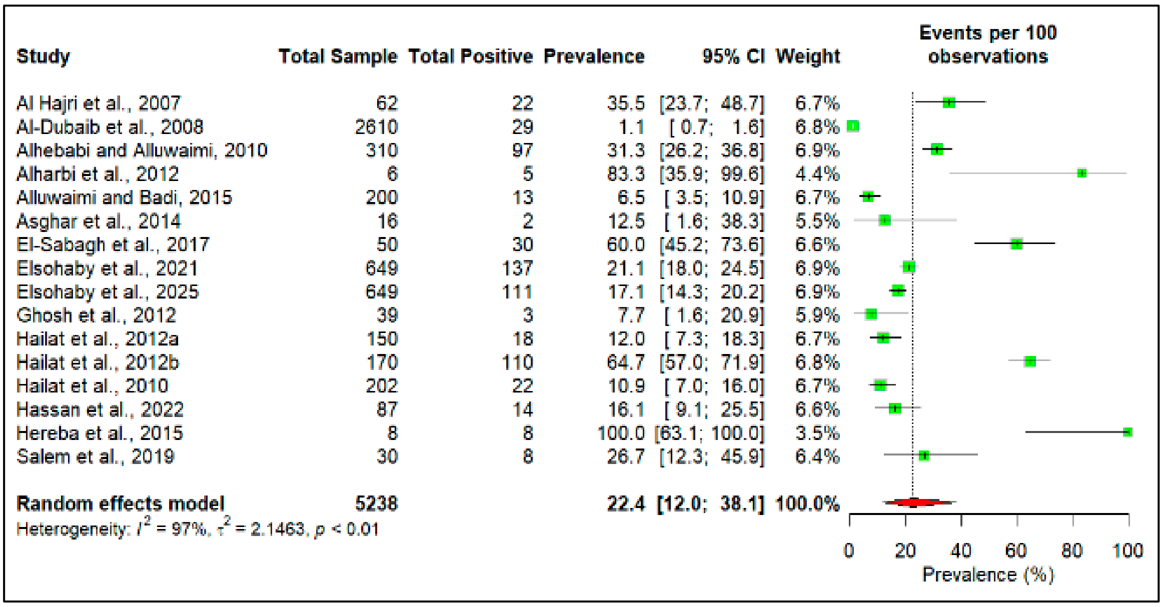

Supplementary Figure 8: Individual animal-level estimated pooled pathogen prevalence of paratuberculosis in the Arabian Peninsula.

2.2.1 Risk factors of pathogen prevalence

2.2.1.1 Country of origin

Study	Total Sample	Total Positive	Prevalence	95% CI	Weight
**Country = Saudi Arabia**					
Al Hajri et al., 2007	62	22	35.5	[23.7; 48.7]	6.7%
Al-Dubaib et al., 2008	2610	29	1.1	[0.7; 1.6]	6.8%
Alhebabi and Alluwaimi, 2010	310	97	31.3	[26.2; 36.8]	6.9%
Alharbi et al., 2012	6	5	83.3	[35.9; 99.6]	4.4%
Alluwaimi and Badi, 2015	200	13	6.5	[3.5; 10.9]	6.7%
Asghar et al., 2014	16	2	12.5	[1.6; 38.3]	5.5%
El-Sabagh et al., 2017	50	30	60.0	[45.2; 73.6]	6.6%
Elsohaby et al., 2021	649	137	21.1	[18.0; 24.5]	6.9%
Elsohaby et al., 2025	649	111	17.1	[14.3; 20.2]	6.9%
Ghosh et al., 2012	39	3	7.7	[1.6; 20.9]	5.9%
Hereba et al., 2015	8	8	100.0	[63.1; 100.0]	3.5%
Salem et al., 2019	30	8	26.7	[12.3; 45.9]	6.4%
**Random effects model**	**4629**		**23.1**	**[10.2; 44.3]**	**73.1%**
Heterogeneity: $I^2 = 97\%$, $\tau^2 = 2.6480$, $p < 0.01$					
**Country = Jordan**					
Hailat et al., 2012a	150	18	12.0	[7.3; 18.3]	6.7%
Hailat et al., 2012b	170	110	64.7	[57.0; 71.9]	6.8%
Hailat et al., 2010	202	22	10.9	[7.0; 16.0]	6.7%
**Random effects model**	**522**		**24.0**	**[5.2; 64.3]**	**20.3%**
Heterogeneity: $I^2 = 98\%$, $\tau^2 = 2.3243$, $p < 0.01$					
**Country = Iraq**					
Hassan et al., 2022	87	14	16.1	[9.1; 25.5]	6.6%
**Random effects model**	**5238**		**22.4**	**[12.0; 38.1]**	**100.0%**
Heterogeneity: $I^2 = 97\%$, $\tau^2 = 2.1463$, $p < 0.01$					
Test for subgroup differences: $\chi^2_2 = 0.78$, df = 2 ($p = 0.68$)					

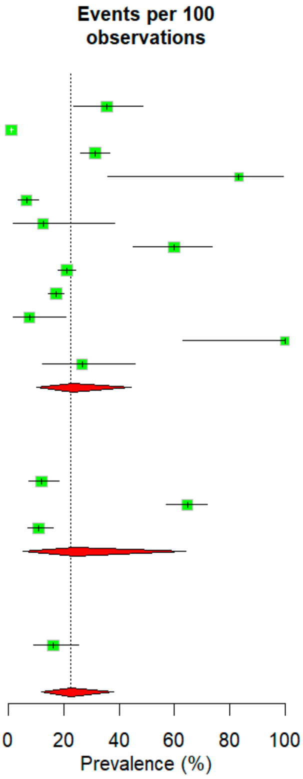

Supplementary Figure 9: Individual animal-level estimated pooled pathogen prevalence of paratuberculosis based on country of origin.

2.2.1.2 Ruminant type

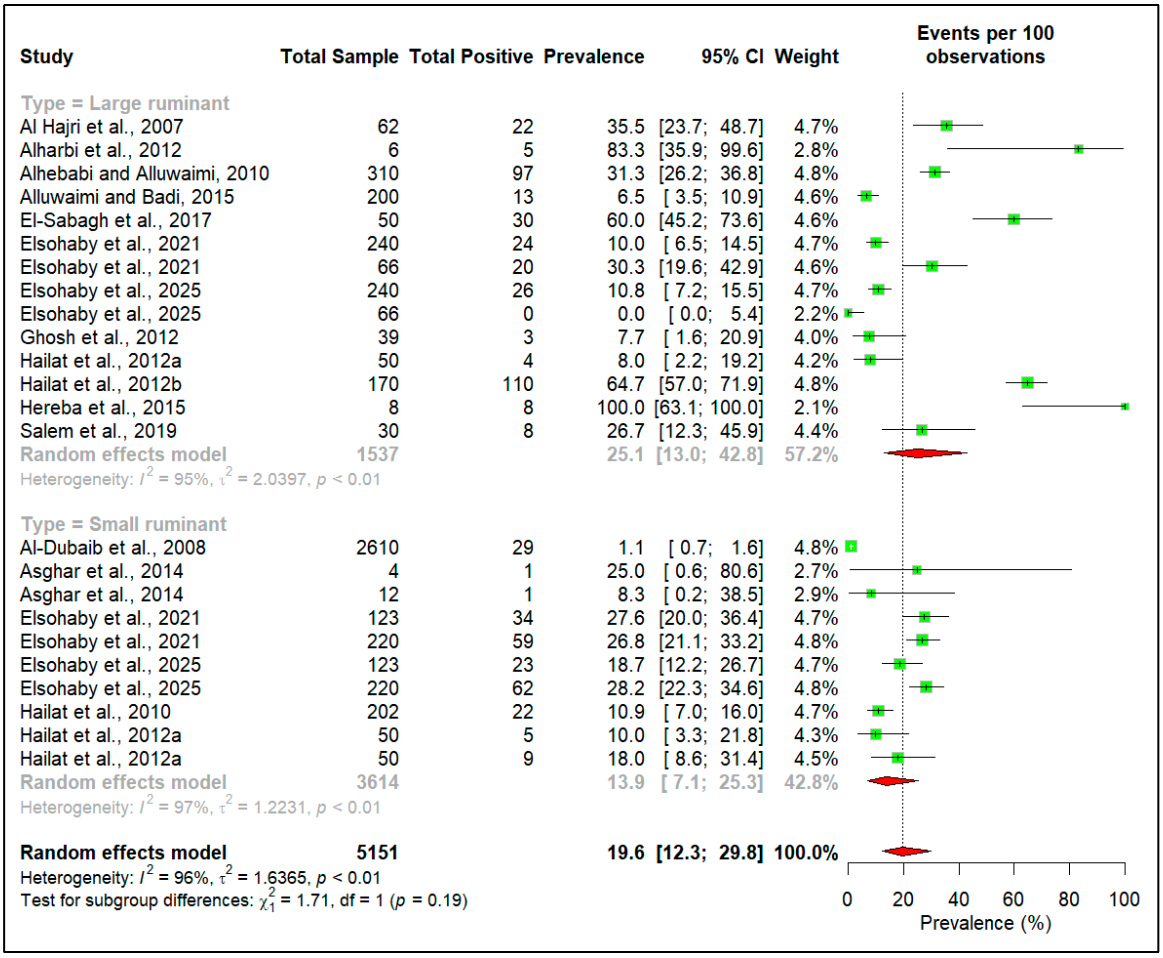

Supplementary Figure 10: Individual animal-level estimated pooled pathogen prevalence of paratuberculosis based on ruminant type.

2.2.1.3 Species

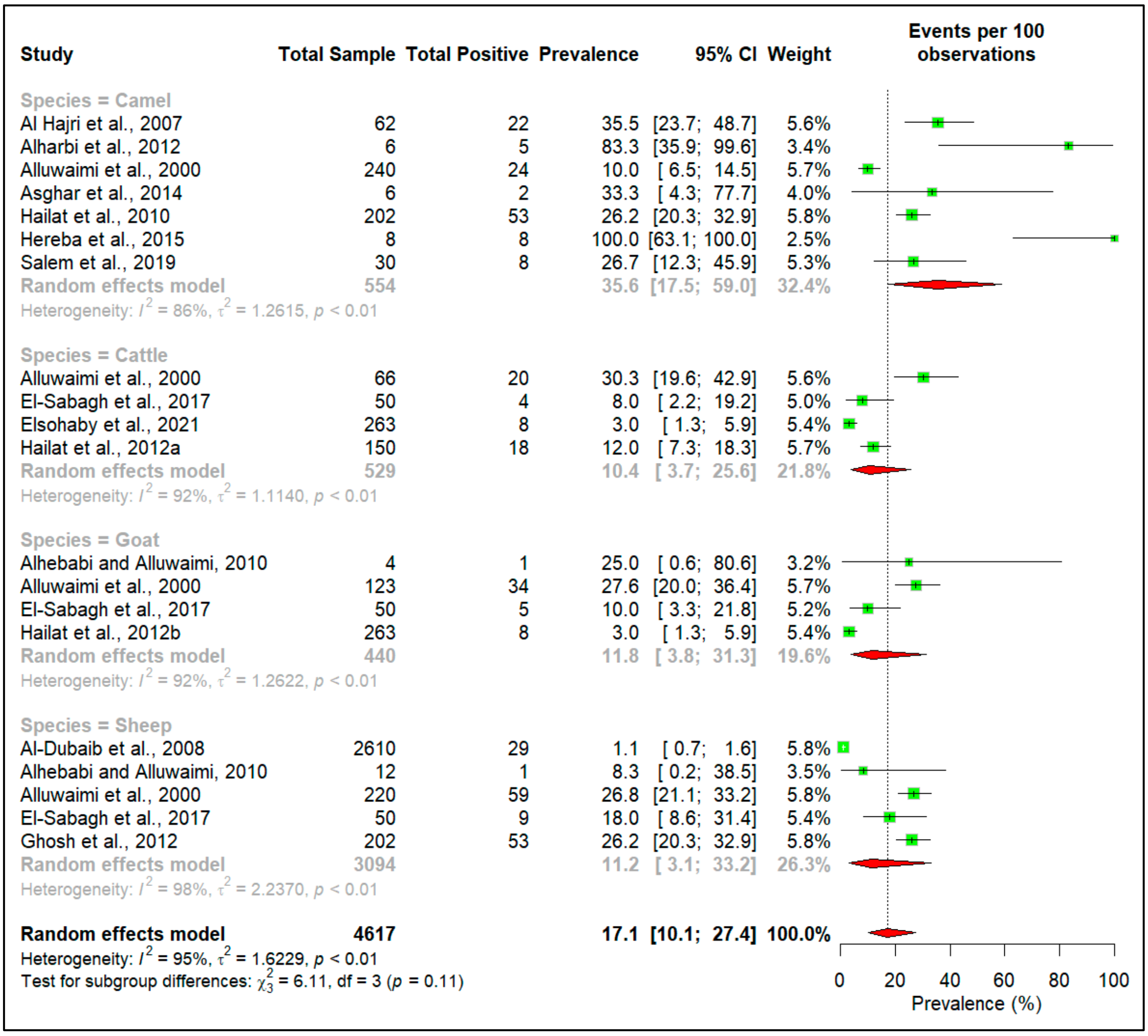

Supplementary Figure 11: Individual animal-level estimated pooled pathogen prevalence of paratuberculosis based on ruminant species.

2.2.1.4 Sex

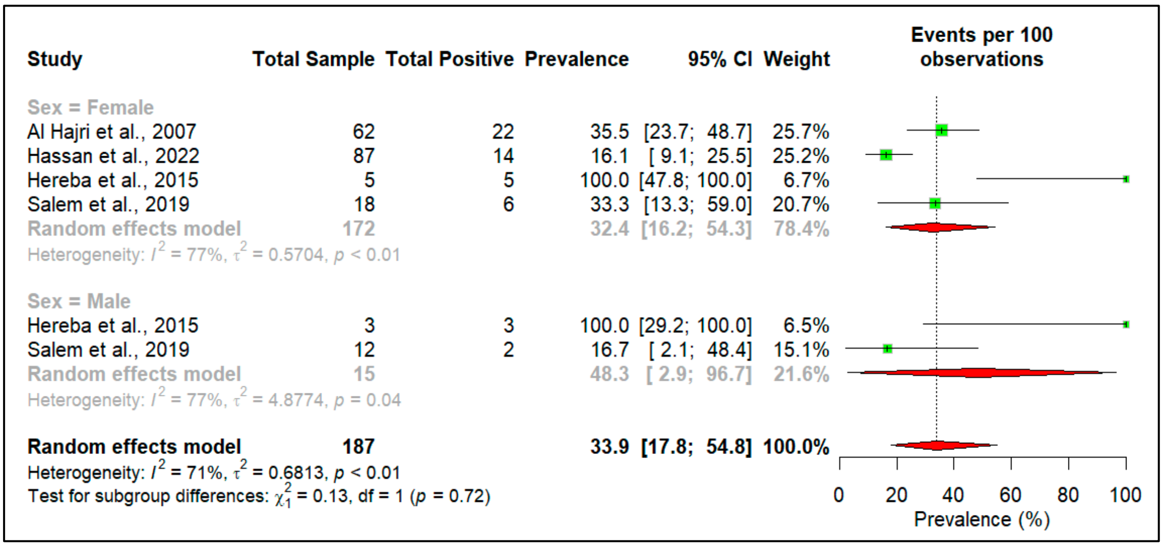

Supplementary Figure 12: Individual animal-level estimated pooled pathogen prevalence of paratuberculosis based on ruminant sex.

2.2.1.5 Age

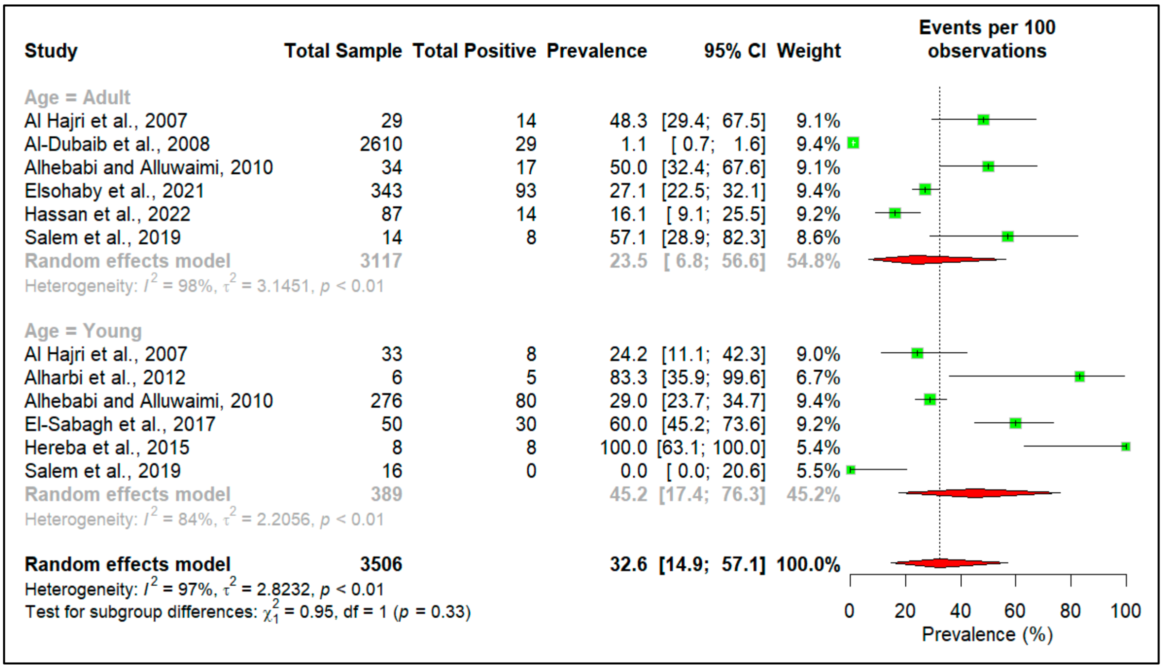

Supplementary Figure 13: Individual animal-level estimated pooled pathogen prevalence of paratuberculosis based on ruminant age.
